# Biological Performances of Plasmonic Biohybrids Based on Phyto-Silver/Silver Chloride Nanoparticles

**DOI:** 10.3390/nano11071811

**Published:** 2021-07-12

**Authors:** Yulia Gorshkova, Marcela-Elisabeta Barbinta-Patrascu, Gizo Bokuchava, Nicoleta Badea, Camelia Ungureanu, Andrada Lazea-Stoyanova, Mina Răileanu, Mihaela Bacalum, Vitaly Turchenko, Alexander Zhigunov, Ewa Juszyńska-Gałązka

**Affiliations:** 1 Joint Institute for Nuclear Research, Frank Laboratory of Neutron Physics, Joliot-Curie, 6, 141980 Dubna, Russia; Yulia.Gorshkova@jinr.ru (Y.G.); gizo.bokuchava@jinr.ru (G.B.); turchenko@jinr.ru (V.T.); 2 Institute of Physics, Kazan Federal University, 16a Kremlyovskaya Street, 420008 Kazan, Russia; 3 Department of Electricity, Solid-State Physics and Biophysics, Faculty of Physics, University of Bucharest, 405 Atomistilor Street, P.O. Box MG-11, 077125 Magurele, Romania; raileanumina@gmail.com; 4 General Chemistry Department, Faculty of Applied Chemistry and Materials Science, University “Politehnica” of Bucharest, 1-7, Polizu Street, 011061 Bucharest, Romania; nicoleta.badea@gmail.com (N.B.); ungureanucamelia@gmail.com (C.U.); 5 National Institute for Lasers, Plasma and Radiation Physics, 409 Atomistilor Street, 077125 Magurele, Romania; andrada@infim.ro; 6 Department of Life and Environmental Physics, Institute for Physics and Nuclear Engineering, Horia Hulubei National, Reactorului, 30, 077125 Magurele, Romania; bmihaela@nipne.ro; 7 Department of Crystal Growth Laboratory, South Ural State University, 76, Lenin Aven., 454080 Chelyabinsk, Russia; 8 Institute of Macromolecular Chemistry AS CR, Heyrovskeho nam. 2, 162 06 Prague 6, Czech Republic; zhigunov@imc.cas.cz; 9 Institute of Nuclear Physics, Polish Academy of Sciences, ul. Radzikowskiego 152, 31-342 Krakow, Poland; ewa.juszynska-galazka@ifj.edu.pl

**Keywords:** silver/silver chloride nanoparticles, “green” synthesis, chlorophyll-*a*-labeled bio-inspired membranes, biohybrids, antioxidant, antibacterial, and antiproliferative activities

## Abstract

Silver/silver chloride nanoparticles (Ag/AgClNPs), with a mean size of 48.2 ± 9.5 nm and a zeta potential value of −31.1 ± 1.9 mV, obtained by the *Green Chemistry* approach from a mixture of nettle and grape extracts, were used as “building blocks” for the “green” development of plasmonic biohybrids containing biomimetic membranes and chitosan. The mechanism of biohybrid formation was elucidated by optical analyses (UV–vis absorption and emission fluorescence, FTIR, XRD, and SAXS) and microscopic techniques (AFM and SEM). The aforementioned novel materials showed a free radical scavenging capacity of 75% and excellent antimicrobial properties against *Escherichia coli* (IGZ = 45 mm) and *Staphylococcus aureus* (IGZ = 35 mm). The antiproliferative activity of biohybrids was highlighted by a therapeutic index value of 1.30 for HT-29 cancer cells and 1.77 for HepG2 cancer cells. At concentrations below 102.2 µM, these materials are not hemolytic, so they will not be harmful when found in the bloodstream. In conclusion, hybrid systems based on phyto-Ag/AgClNPs, artificial cell membranes, and chitosan can be considered potential adjuvants in liver and colorectal cancer treatment.

## 1. Introduction

Nowadays, the use of natural resources and, in particular, plants has experienced a great importance, thanks to the impressive number of bioactive ingredients with a strong beneficial impact on human health. The intensive development of new hybrid lipid–nanoparticle complexes, including inorganic nanoparticles (NPs), lipid assemblies, and lipid–polymer complexes, is caused by numerous potential biomedical applications [1,2]. Soy lecithin liposomes represent a promising drug delivery system, especially in the fight against serious illnesses such as tuberculosis and malaria [3]. Modification of the surface of liposomes changes their physicochemical and biophysical properties that are able to overcome the limitations of conventional liposomes. An important requirement for the development of modified liposomes for drug delivery is the stability of such systems, excluding their premature fusion with each other, on the one hand, and their activation upon reaching a target such as viruses or bacteria, on the other. Polymers play an important role in stabilizing liposomes. An alternative method to develop hybrid nanomaterials for biomedical and pharmaceutical applications is the modification of the liposomes using the natural-derived polymer chitosan (CTS), which is recognized as a universal biomaterial because of its nontoxicity, low allergenicity, biocompatibility, and biodegradability [4]. Chitosan is a chitin-derived polymer with a linear and semi-crystalline structure bearing three functional groups on its main backbone: one amino (NH_2_) group and two hydroxyl (OH) groups [5]. This positively charged polysaccharide [6] was used to develop chitosan–silver composites [7] for wound-dressing applications [8] due to its low cytotoxicity and high antimicrobial properties. Additionally, some successful results with chitosan-coated lecithin liposomes and phytosomes were reported by the research teams of Filipović-Grcić [9] and Barbinta-Patrascu [10], respectively. An alternate strategy is the stabilization of the liposomes via surface-bound inorganic metal nanoparticles, for example with silver nanoparticles (AgNPs) [11]. AgNPs have the advantage of possessing a great antibacterial effect [12]. Other authors reported the improved stability, compatibility, and antibacterial properties of one-step synthesis of AgNPs-stabilized liposomes compared to AgNPs alone [13]. 

In recent decades, “green” syntheses of AgNPs from plant extracts have also been developed to reduce the toxicity of solvents and stabilized agents. Generally, the synthesis of nanoparticles from plant extracts has the advantage of being a low-cost, environmentally friendly, rapid, and facile method. Phytofabricated eco-friendly silver nanoparticles showed higher bactericidal, antioxidant, and anti-inflammatory activities [14]. In addition, several authors reported the fabrication of silver chloride nanoparticles (AgClNPs) or hybrid Ag/AgClNPs from plant extracts [15,16,17,18,19,20,21]. Such Ag/AgCl plasmonic hybrids have antibacterial properties with low cytotoxicity, which may be attributed to the solubility equilibrium of Ag^+^ controlled by AgCl that allows for a low Ag^+^ level to be released into the environment [18,19]. Silver/silver chloride nanoparticles were also synthesized by Kota et al. [21] by using aqueous leaf extract of *Rumex acetosa*; these nanoparticles demonstrated good antimicrobial and antioxidant properties, as well as cytotoxicity against the tested human osteosarcoma cell lines.

The challenge of our study is to demonstrate that silver/silver chloride nanoparticles fabricated according to a “green” protocol, as well as the biohybrids based on Ag/AgClNPs, have a high potential for biomedicine. The aim of this study was to improve the antimicrobial, antioxidant, and anticancer properties of Ag/AgClNPs, in combination with artificial cell membranes, and chitosan. In this work, an aqueous extract of a mixture of nettles and grapes to create hybrid nanoparticles was used. Three types of silver-based biohybrids with and without chitosan were produced. These nettle and grape extracts contain biocompounds with excellent biological value (such as antioxidant and antibacterial properties) [22]. Physicochemical properties of the obtained phyto-based materials were evaluated through optical (UV–vis, fluorescence emission, and FTIR spectroscopy), structural (XRD and SAXS), and microscopic (AFM and SEM) investigations. The physical stability of the suspensions of the developed materials was estimated through zeta potential measurements. Moreover, the antioxidant, antibacterial, and antiproliferative activities were tested to assess the bioperformances of our biodeveloped materials.

## 2. Materials and Methods

### 2.1. Materials

Tris (hydroxymethylaminomethane base), HCl, H_2_O_2_, luminol (5-amino-2,3-dihydro-phthalazine-1,4-dione), silver nitrate (AgNO_3_), KH_2_PO_4_, and Na_2_HPO_4_ were supplied from Merck (Darmstadt, Germany). Standard hemoglobin, soybean lecithin, NaCl, Drabkin reagent, ethidium homodimer-1 (EthD-1), and acridine orange (AO) were purchased from Sigma-Aldrich (Darmstadt, Germany). The yeast extract was supplied from BioLife, and the agar was obtained from Fluka (Switzerland). Chlorophyll *a* was extracted in our laboratory from fresh spinach leaves according to the method of Strain and Svec [23]. The nettle leaves and the grapes were acquired from a local market. 

Bacteria culture test. Antimicrobial activity of the samples was tested against pathogenic Gram (-) bacteria *Escherichia coli* ATCC 8738 and Gram (+) bacteria *Staphylococcus aureus* ATTC 25923. These bacterial cultures were maintained at 4 °C. All chemicals used for antibacterial investigations were purchased from VWR (Darmstadt, Germany). 

Cell culture and reagents. Three different cell lines were used for the in vitro studies. Human fibroblast BJ cells (ATCC CRL-2522, Manassas, VA, USA) and human colorectal adenocarcinoma HT-29 cells (ATCC, Manassas, VA, USA) were grown in Minimal Essential Medium (MEM) supplemented with 2 mM L-Glutamine, 10% fetal calf serum (FCS), 100 units/mL of penicillin, and 100 µg/mL of streptomycin at 37 °C in a humidified incubator under an atmosphere containing 5% CO_2_. Human hepatocarcinoma HepG2 cells (ATCC, Manassas, VA, USA) were grown in DMEM supplemented with similar reagents. All cell cultivation media and reagents were purchased from Biochrom AG (Berlin, Germany). Drabkin reagent and standard hemoglobin were purchased from Sigma-Aldrich (Darmstadt, Germany).

### 2.2. Preparation of Nanosilver-Based Biohybrids

#### 2.2.1. Phytogeneration of Silver/Silver Chloride Nanoparticles

Two types of aqueous extracts of nettle leaves and grapes were prepared as described in [22]. A mixture of (0.24 mL of nettle extract + 0.06 mL of grape extract) was added to a volume of 30 mL of 1 mM AgNO_3_ aqueous solution, under continuous magnetic stirring, and then placed at room temperature for one day. This suspension was diluted 1.62 times with phosphate-buffered saline (PBS, KH_2_PO_4_/Na_2_HPO_4_/NaCl, pH 7.4) and furthermore subjected to an ultrasound treatment in an ultrasonic bath (BRANSON 1210, Marshall Scientific, Hampton, NH, USA) for 30 min. The resulting Ag/AgClNPs were diluted in the biodispersant PBS to a final silver content of 0.61 mM.

#### 2.2.2. Preparation of Artificial Cell Membranes 

Artificial cell membranes labeled with Chl*a* were obtained by the hydration of soybean lecithin thin as previously described [24]. The resulting liposome suspensions were divided into two parts: without and with chitosan. An acidic solution (0.4% acetic acid *v*/*v* in distilled water) of 1% (*w*/*v*) chitosan (CTS) was added to a liposomal suspension, to a final CTS concentration of 0.01% (*w*/*v*); this mixture was then strongly stirred for 15 min (200 rpm, VIBRAX stirrer, Milian, OH, USA). These two types of biological entities were further diluted in PBS to a final lipid concentration of 0.34 mg/mL.

#### 2.2.3. Bottom-up “Green” Design of Plasmonic Biohybrids

Three types of hybrid silver-based systems were prepared based on suspensions of Ag/AgClNPs and liposomes, with and without chitosan solution, according to Table 1 (see Samples P4–P6) by using strong stirring (VIBRAX stirrer, Milian, OH USA 200 rpm) for 15 min and ultrasound treatment in an ultrasonic bath (BRANSON 1210, Marshall Scientific, Hampton, NH, USA) for 30 min. 

All these biohybrids were diluted in PBS to a final lipid concentration of 0.34 mg/mL and with a final silver content of 0.61 mM.

**Figure 1:**
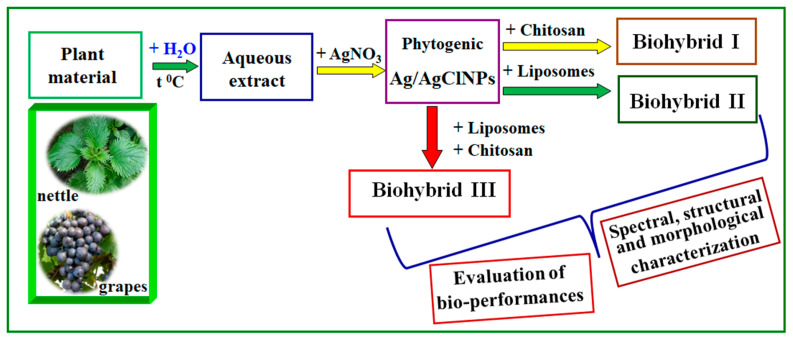
Schematic representation of the “green” development of biohybrids generated from nettle and grape extracts.

Figure 1 shows a diagram representing the “green” development of biohybrids generated from the aqueous extract of a mixture of nettle and grape extracts. 

### 2.3. Physicochemical and Biological Characterization of the Developed Bioentities

#### 2.3.1. Spectral and Morphological Characterization

The absorption spectra of the samples were recorded (at the resolution of 1 nm) from 200 to 800 nm on a double-beam Jasco V-670 UV–vis–NIR spectrophotometer (Jasco, Tokyo, Japan). 

The fluorescence emission spectra of chlorophyll-*a*-based samples were collected using a LS55 Perkin Elmer fluorescence spectrometer (Waltham, MA, USA) in the wavelength range of 600–800 nm by illuminating the samples with a 430 nm excitation light. 

Fourier-transform infrared (FTIR) spectra of initial components AgNPs, liposomes, and biohybrid complexes were recorded on an Excalibur FTS–3000 FTIR spectrometer (Bio-Rad, Munich, Germany), in the wavenumber range of 4000–500 cm^−1^. Each spectrum was averaged over 128 scans, and the spectral resolution was 0.4 cm^−1^ at a constant temperature of 40 ± 1 °C. The samples were thin films, placed between two ZnSe window discs. During the recorded spectra, the spectrometer was purged with dry nitrogen. For all obtained spectra, the baseline correction and normalization were applied.

For atomic force microscopy (AFM) experiments, the sample solutions (50 μL aliquots) were deposited onto freshly cleaved 15 mm × 15 mm mica squares, left to adsorb for 3 min, and rinsed with Millipore water added dropwise to remove redundant samples. The surface was air dried (3 h) in a dust-free enclosure at room temperature, then imaged using AFM. Atomic force microscopy NTEGRA PRIMA was provided by NT-MDT Spectrum Instruments (Zelenograd, Russia). AFM images were recorded in tapping mode with commercial NSG01 tips of a 10 nm curvature radius (NT-MDT Spectrum Instruments, Zelenograd, Russia) at room temperature. Images were taken continuously at a scan rate of 0.3−0.5 Hz. Both the height image and the phase image were recorded. The images were flattened using the NT-MDT Spectrum Instruments Image Analysis P9 software.

The surface morphology of the samples presented in this paper was analyzed using a scanning electron microscope (SEM), a FEI Inspect Model S50 apparatus (Hillsboro, OR, USA). The apparatus is equipped with a secondary electrons (SE) detector. The SEM images were obtained at a 10 mm working distance, a 10 kV acceleration voltage, and for 50 up to 50,000× magnifications. Before SEM investigations, all the samples were coated with a thin Au layer (~10 nm). The Au layer was obtained using a sputtering Cressington 108 auto sputter coater apparatus (Cressington Scientific Instruments UK, Watford, England (UK)), equipped with a Cressington MTM-20 thickness controller. The ImageJ program was used to estimate the size of the developed materials from the magnified SEM images.

The hydrodynamic diameters (Zav) of samples were measured by the dynamic light scattering (DLS) technique (Zetasizer Nano ZS, Malvern Instruments Ltd., Worcestershire, U.K.), at a scattering angle of 90° by the Stokes–Einstein equation. The mean values were calculated from three individual experiments, so the average values (±standard deviation, SD) were further reported. The polydispersity indexes (PdI—the indicator of the width of the particle size distribution) were also determined from 3 individual measurements using intensity distribution.

Zeta potential (ξ) measurements were carried out at 25 °C in triplicate, and the mean values were reported on Malvern Zetasizer Nano ZS (Malvern Instruments Inc., Worcestershire, UK) by measuring, in an electric field, the electrophoretic mobility of the prepared samples.

The crystallographic structure and phase composition of the sample series P3–P6 containing Ag/AgClNPs were examined by X-ray diffraction (XRD) by using an EMPYREAN diffractometer (PANalytical, Almelo, The Netherlands) with Cu-Kα incident radiation. Samples P3–P6 were extracted from the PBS solution by centrifuging (15,000× *g*, 30 min) at 4 °C. The separated upper layer (supernatant) was removed. The liquid sediment was placed on quartz glass (2.5 cm × 2.5 cm) and was evaporated in a vacuum chamber for 12 h at room temperature. The XRD spectra were measured with an exposition time of 12 h for each sample. The average sizes of nanoparticles for each phase were estimated using the Scherrer equation:

(1) $$D=\frac{K\lambda }{\beta \cos\vartheta },$$

where *D* is the average crystallite size, *K* is a dimensionless shape factor close to unity, λ is the X-ray wavelength, θ is the Bragg angle, and β is the peak width at half the maximum intensity (FWHM) after subtracting the instrumental line broadening.

SAXS experiments were performed using a pinhole camera (MolMet, Rigaku, Japan, modified by SAXSLAB/Xenocs) attached to a microfocused X-ray beam generator (Rigaku MicroMax 003) operating at 50 kV and 0.6 mA (30 W). The camera was equipped with a vacuum version of the Pilatus 300 K detector. Calibration of primary beam position and sample-to-detector distances was performed using a AgBehenate sample. For the measurements, samples were sealed into borosilicate capillaries. Two experimental setups were used to cover the *q* range of 0.005–0.5 Å^−1^. The scattering vector, *q*, is defined as *q* = *(4π/λ)sin(Θ)*, where *λ* is the wavelength and 2*Θ* is the scattering angle. Homemade software based on the PyFAI Python library [25] was used for data reduction.

#### 2.3.2. In Vitro Antioxidant Activity

The chemiluminescence (CL) technique (Turner Design TD 20/20 USA Chemiluminometer) was used to evaluate the antioxidant properties of the samples by using a free radical generator system based on 10^−5^ M luminol and 10^−5^ M H_2_O_2_ in TRIS-HCl buffer (pH 8.6). The in vitro antioxidant activity (AA%) of each sample was calculated, in triplicate, as:

AA = [(I_0_ − I)/I_0_]∙100%, (2)

where I_0_ and I are the maximum CL intensities at t = 5 s for the control (i.e., the reaction mixture without the sample) and for each sample, respectively [26].

#### 2.3.3. Antibacterial Activity

Bacteria stock cultures (*Staphylococcus aureus* and *Escherichia coli*) were subcultured onto Luria Bertani Agar acc. Miller (LBA) plates at 37 °C. To evaluate the antibacterial properties of the tested samples, the antibacterial activity was determined by the agar well diffusion method as previously described in [27]. Briefly, the LBA surface was inoculated by spreading a volume of the bacteria inoculum. Using a sterile Durham tube 6 mm in diameter, the wells were made and inoculated with 50 μL of each sample; then, the Luria Bertani Agar plates were incubated at 37 °C for 24 h. The antimicrobial agent diffused into the LBA and inhibited the growth of the test bacteria, and then the diameters of the inhibition growth zones (IGZs) were measured.

#### 2.3.4. Cell Viability

The biocompatibility of the biohybrids was evaluated using MTT (3-(4,5-dimethylthiazol-2-yl)-2,5-diphenyltetrazolium bromide) tetrazolium reduction assay as follows. Cells (BJ, HT-29, and HepG2) were seeded in 96-well plates and cultured for 24 h in medium. The next day, the medium was changed, and different concentrations of biohybrids were added for 24 h. Cells grown only in medium were used as negative control. Following incubation, the medium was changed, and 1 mg/mL of MTT solution was added to each well and incubated for an additional 4 h at 37 °C. Finally, the medium was collected, and DMSO was used to dissolve the insoluble formazan product. The absorbance of the samples was recorded at 570 nm using a Mithras 940 plate reader (Berthold, Germany). The data were corrected for the background, and the percentage of viable cells was obtained using the following equation:

% Viable cells = [(A_570_ of treated cells)/(A_570_ untreated cells)]·100%, (3)

The NP concentration that reduced the viability of the cells by half (IC_50_) was obtained by fitting the data with a logistical sigmoidal equation using the software Origin 8.1 (Microcal Inc., Northampton, MA, USA.). The therapeutic index (TI) was calculated as the ratio of the dose that produces toxicity to the dose needed to produce the desired therapeutic response [28].

#### 2.3.5. Evaluation of Cellular Morphology

Cells were grown on coverslips and treated for 24 h with two different concentrations for the biohybrids. Afterwards, the cells were washed with PBS, fixed for 15 min with 3.7% formaldehyde dissolved in PBS, and washed again with PBS buffer. Sequentially, cells were stained with 20 μg/mL AO solution for 15 min, then immediately washed with PBS, followed imaging using an Andor DSD2 Confocal Unit (Andor, Belfast, Northern Ireland), mounted on an Olympus BX-51 epifluorescence microscope (Olympus, Hamburg, Germany), equipped with a 40× objective and an appropriate filter cube (excitation filter, 466/40 nm; dichroic mirror, 488 nm; and emission filter, 525/54 nm).

#### 2.3.6. Hemocompatibility

The hemolytic activity of the new hybrids was determined using an adapted protocol based on the ASTM F 756-00 standard previously described [29]. Briefly, fresh blood was collected on heparin from healthy volunteers and diluted with PBS to a final hemoglobin concentration of ~10 mg/mL. The blood was incubated with the highest concentration of the samples for 4 h at 37 °C under constant shaking. Finally, the supernatant was collected and mixed with an equal amount of Drabkin reagent (Sigma-Aldrich, Darmstadt, Germany). After 15 min, the absorbance of the samples was read at 570 nm using a plate reader. As negative and positive controls, the human red blood cells (hRBCs) in PBS and distilled water, respectively, were used. The experimental values were corrected for background dilution factors and used to calculate the percentage of hemolysis (i.e., hemolytic index), according to the equation:

% Hemolysis = (A_S_/A_T_) 100%, (4)

where A_S_ is the corrected absorbance of the hemoglobin released in the supernatant after treatment with nanoparticles, and A_T_ is the corrected absorbance of the total released hemoglobin.

#### 2.3.7. Statistical Analysis

Unless stated otherwise, all data were expressed as the mean value ± standard deviation of three individual experiments. Statistical significance was estimated using the *Student’s t-test* (Microsoft Excel 2010) to determine the significant differences among the experimental groups, and values of *p* < 0.05 were considered statistically significant.

## 3. Results and Discussion

### 3.1. Optical Characterization of the Developed Materials

The developed bionanosilver-based hybrid materials were optically characterized by UV–vis absorption, fluorescence emission, and FTIR spectroscopy, in order to acquire deep insights about the nanobiointeraction between components of biohybrids and also about the biohybrids’ formation. Chlorophyll *a* (Chl*a*) incorporated in artificial cell membranes was used as a spectral biosensor to monitor the formation of plasmonic biohybrids.

In the absorption spectra of the samples containing artificial cell membranes, the spectral signature of Chl*a* at ~669 nm was observed. Moreover, the SPR band at ~431 nm, characteristic for Ag/AgClNP formation, was identified in the UV–vis absorption spectra of the silver-based materials (see Figure S1 in the Supplementary Materials).

The formation of plasmonic biohybrids was also confirmed by fluorescence emission spectra of Chl*a*-containing samples (λ_excitation_ = 430 nm), which underwent considerable emission quenching (see Figure S2 in the Supplementary Materials) at the interaction between the porphyrinic ring of Chl*a* and the surface of phyto-Ag/AgClNPs.

FTIR spectra allow for the identification of functional groups present in the components of the systems under investigation. The observation of the vibration spectrum of the biohybrid nanocomplexes allows evaluating the type of interaction that occurs between the Ag/AgClNPs and lecithin liposomes in the presence of chitosan or without it, as the vibrations of the atoms involved in this interaction can suffer changes in frequency and intensity (see Figure S3). These FTIR results indicate the involvement of hydroxyl and carbonyl groups (belonging to polyphenols and proteins, respectively) in the generation of biohybrids P5 and P6.

More details regarding the optical characterization of the developed materials are presented in the Supplementary Materials.

### 3.2. Estimation of Particle Size and Zeta Potential of the Obtained Biohybrids

The mean hydrodynamic diameters of the developed particles were estimated by DLS measurements (see Figure S4 in the Supplementary Materials). As observed, the addition of chitosan to Liposomes, Ag/AgClNPs, and Ag/AgClNPs–Liposomes resulted in a size increase. This aspect will be further confirmed by SAXS experiments and microscopic studies.

**Figure 2:**
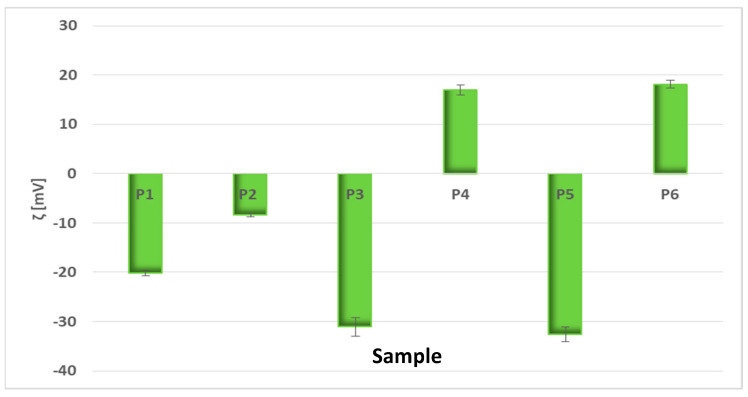
Evaluation of the physical stability of the obtained bio-based materials.

The physical stability of the suspensions of the developed particles is related to the particle electrical charge, which was quantified by zeta potential (ξ) measurements via the electrophoretic mobility of the particles in an electric field [30,31]. Great ξ magnitude is indicative of a stable system [32,33], being related to high electrical interparticle repulsion due to the surface charge, thus indicating the high stability of the suspension. Figure 2 displays the zeta potential values of the bio-based materials developed in this study.

Chlorophyll-labeled artificial cell membranes exhibited moderate stability (ξ_P1_ = −20.17 ± 0.49 mV), and after chitosan addition, the zeta potential value increased to −8.45 ± 0.27 mV (for P2) due to the positive charge of amino functional groups in chitosan. “Green” synthesized silver nanoparticles alone and embedded in biomimetic membranes registered more accentuated electronegative values (ξ_P3_ = −31.1 ± 1.9 mV and ξ_P5_ = −32.57 ± 1.5 mV, respectively), thus good stability. In the presence of chitosan, these values shifted to positive values: ξ_P4_ = +17.5 ± 1.07 mV and ξ_P6_ = +18.1 ± 0.79 mV, respectively. The surface positivity of “green” developed nanoparticles can be attributed to their stabilization with the positively charged chitosan [34].

### 3.3. Structural Characterization of the Biohybrid Complexes

XRD and SAXS analyses were performed to investigate the structure of the obtained materials.

#### 3.3.1. X-ray Diffraction

**Figure 3:**
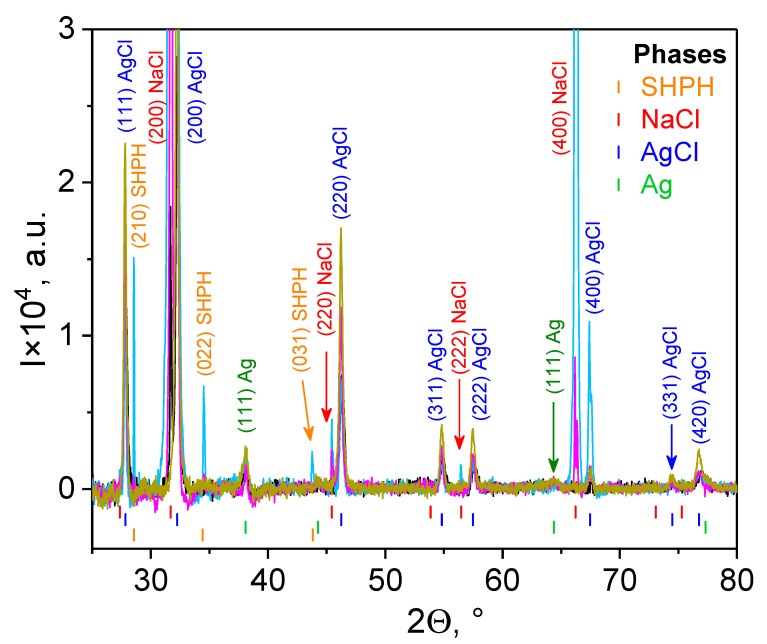
XRD patterns for samples with hybrid Ag/AgCl nanoparticles phytogenerated from nettles and grapes: P3—Ag/AgClNPs (light blue line), P4—Ag/AgClNPs–CTS (black line), P5—Ag/AgClNPs–Liposomes (magenta line) and P6—Ag/AgClNPs–Liposomes–CTS (dark yellow line). The identification of XRD peaks was done according to Inorganic Crystal Structure Database (ICSD) files as follows: (1) SHPH—ICSD Entry: 16138; (2) NaCl—ICSD Entry: 28948; (3) AgCl—ICSD Entry: 64734; (4) Ag—ICSD Entry: 64994.

Phase characterization and chemical composition of the liquid sediments, separated from the PBS solution by centrifugation at 4 °C, followed by evaporation at room temperature, were performed for all samples, which included hybrid Ag/AgCl nanoparticles phytogenerated from nettles and grapes, i.e., for Samples P3–P6 (Figure 3). All XRD spectra demonstrate the presence of two phases: Ag and AgCl, which have a face-centered cubic (FCC) structure with a $Fm\bar{3}m$ space group and lattice parameters a_Ag_ = 0.40895 nm and a_AgCl_ = 0.55487 nm, correspondingly. Moreover, it resulted from the peak intensities that the AgCl phase substantially dominated in all the studied systems. This phenomenon can be explained by two reasons. First, we demonstrate that hybrid Ag/AgCl nanoparticles are the result of the synthesis from biocompounds of nettle and grape aqueous extracts (see Section 3.1 and Supplementary Materials), as were observed in the case of the various plant extracts mediated [35,36]. Second, because of the effect of the PBS buffer, when the anions of Cl^−^ bond to Ag^+^, the AgCl phase becomes dominant in the composition of Ag/AgClNPs.

Two phases of NaCl and sodium hydrogen phosphate hydrate (SHPH) are remaining byproducts arising from the PBS buffer. The NaCl phase has a face-centered cubic (FCC) structure with a $Fm\bar{3}m$ space group and a lattice parameter a_NaCl_ = 0.5640 nm. The SHPH phase with chemical formula Na_2_HPO_3_(H_2_O)_5_ has an orthorhombic structure (space group Pmn2_1_) with lattice parameters a = 0.7170 nm, b = 0.6360 nm, and c = 0.9070 nm [37]. The average crystallite sizes of the nanoparticles (D_XRD_) for each phase, estimated using the Scherrer equation (Equation (1)), are collected in Table 2.

#### 3.3.2. SAXS Results

Small-angle X-ray scattering was used to study the structural changes of the Ag/AgCl nanoparticles in the biohybrid complexes P3–P6 in excess of PBS medium at the nanoscale level.

**Figure 4:**
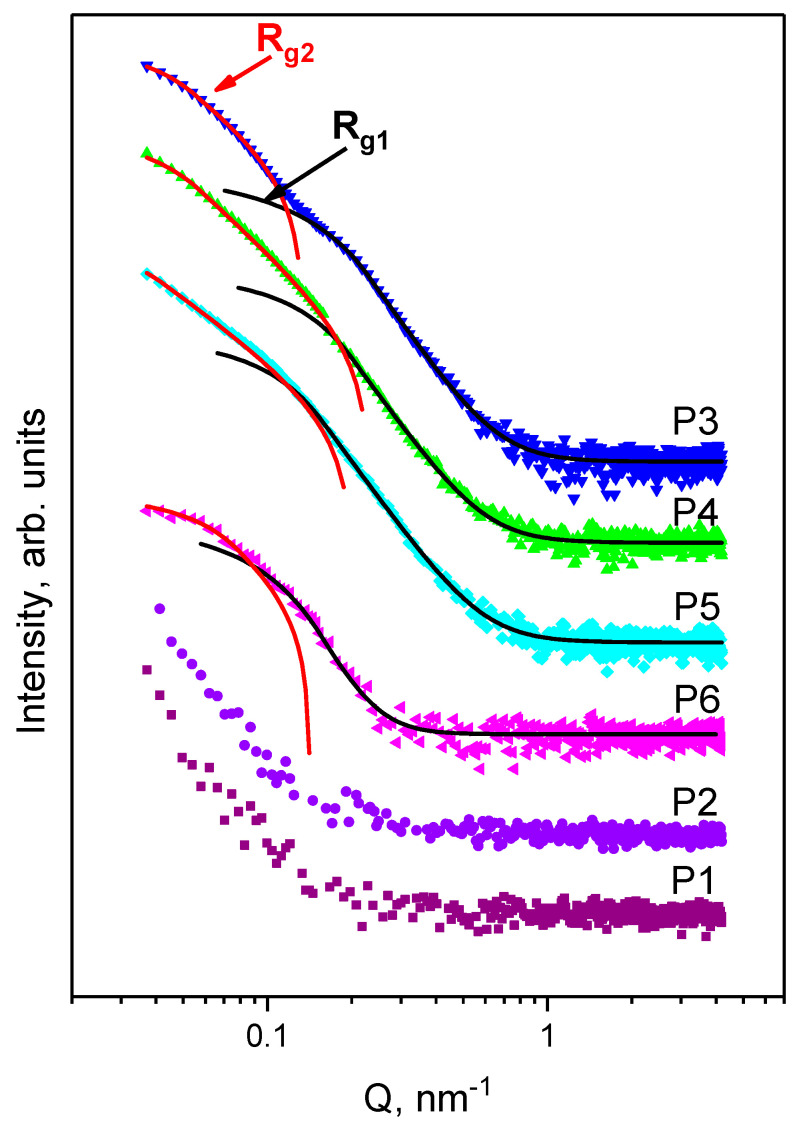
SAXS curves for Samples P1–P6 in excess PBS medium: P1—Lip (purple), P2—Lip–CTS (violet), P3—Ag/AgClNPs (blue), P4—Ag/AgClNPs–CTS (green), P5—Ag/AgClNPs–Lip (cyan) and P6—Ag/AgClNPs–Lip–CTS (magenta). Symbols are experimental data, and lines are fits by the generalized Guinier–Porod model (Equation (1) in the Supplementary Materials). For better visualization, the curves are spaced with a coefficient of 10 relative to each other in the intensity scale from the bottom to the top.

The proposed model (Guinier–Porod) allows for the determination of the size and dimensionality of the objects (see Supplementary Materials). Scattering curves for P3, P4, and P5 are quite similar [38]. The SAXS experimental data for the initial components and their complexes are shown in Figure 4. The obtained parameters from the fitting procedure on the experimental SAXS data are presented in Table 3.

Best fits are presented in Figure 4 as lines. The presence of two structural levels is clearly seen from the SAXS curves. The scattering objects were tentatively divided into two parts: smaller nanoparticles with R_g1_ ranging from 10.1 nm up to 15.7 nm and larger nanoparticles with R_g2_ ranging from 33.7 nm up to 43.4 nm. Based on the dimensionality value(s), we can assume a spherical shape of NPs. Then, the average diameter can be calculated as D_SAXS_ = 2(5/3)^1/2^R_g_. The obtained values are presented in Table 3.

SAXS and XRD results both confirm the presence of two types of nanoparticles in the system: AgNPs and hybrid Ag/AgClNPs. The findings achieved through XRD and SAXS analyses support the results obtained by UV–vis absorption spectroscopy. Thus, the mixture with CTS (Sample P4) increased to a larger diameter by 5 nm, while in the sample with liposomes (Sample P5), we observed an increase by 25 nm. Interestingly, the scattering curve for Sample P6 slightly differs from other complexes with Ag/AgClNPs. The size of the small nanoparticles is identical to the values determined for the previously discussed samples, while the larger nanoparticles are much smaller than observed for Samples P3-P5. Note that the small NPs containing Ag crystallites have a diffusive interface [39], resulting in higher anisotropy objects and thus higher values of the dimensionality parameter. For larger Ag/AgClNPs, the power-law exponent implies that scattering occurs on the mass fractals with a fractal dimension of ~3 [39,40,41,42].

### 3.4. Morphological Characterization of the Developed Bio-Based Materials

The surface characteristics of the biohybrids were studied using AFM and SEM methods.

#### 3.4.1. AFM Analysis of the Developed Materials

**Figure 5:**
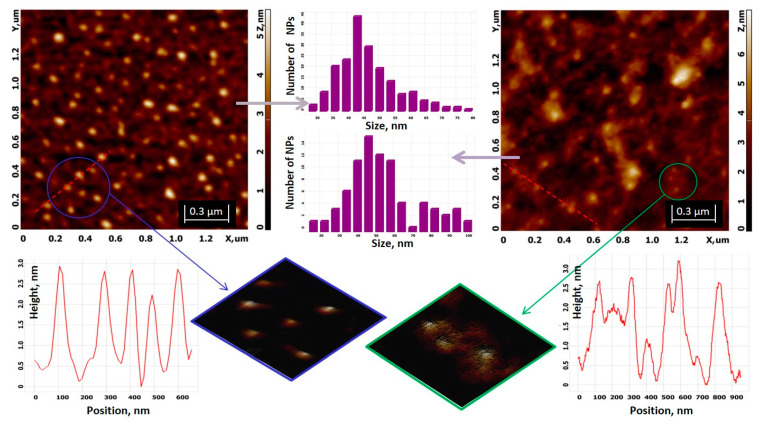
Topologies of Ag/AgClNPs (P3, **left**) and Ag/AgClNPs–CTS Biohybrid I (P4, **right**) with size distributions (in the **middle**) for both systems, obtained using Image Analysis software (version 3.5) (https://nexus.ntmdt.ru/dl_3687; accessed on 4 March 2020). **Bottom** figures (from left to right): height analysis in the selected direction (red dashed line) and magnified 3D visualization of the selected blue area for the P3 system, cropped 3D image of the selected green area, and height analysis in the selected direction (red dashed line) for the P4 system.

AFM topology of the hybrid Ag/AgClNPs is shown in Figure 5 (left). The NPs have a spherical shape, which is in good agreement with the results obtained from the approximation of the SAXS curves (see Figure 4). The average size of Ag/AgClNPs is 48.2 ± 9.5 nm. Silver/silver chloride nanoparticles in the presence of chitosan are involved in the polymer network, as is clearly seen in Figure 5 (right).

Ag/AgClNPs coated with chitosan have an average size of 56.9 ± 19.5 nm. The size distribution includes aggregates ranging in size from 75 to 100 nm. Biohybrid Complex I consists of NPs with a chitosan shell, indicated by the following items: (1) an increase in NP size; (2) an increase in surface roughness (R_a_P3_ = 0.5 nm, R_a_P4_ = 0.7 nm, R_q_P3_ = 0.57 nm, and R_q_P4_ = 0.9 nm); and (3) the appearance of a rough surface of Ag/AgClNPs in the presence of CTS compared to the smooth surface of Ag/AgClNPs without CTS, as can be clearly seen from the height analysis and magnified 3D images for P3 and P4 systems (Figure 5, bottom figures).

Low height is, according to AFM, a flattening effect that occurs during the adsorption of the nanoparticles on mica and their drying. More details regarding the sonication time effect on liposome formation are given in Figure S5 in the Supplementary Materials.

**Figure 6:**
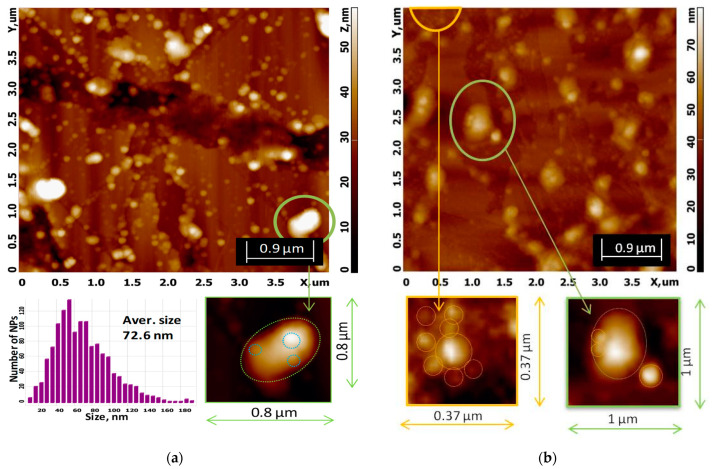
(**a**) AFM image of Ag/AgClNPs–Liposomes (P5—Biohybrid II) with the size distribution of the “free” silver/silver chloride nanoparticles. The cropped green fragment illustrates the association of Ag/AgClNPs with chlorophyll-a-labeled soybean lecithin liposomes. (**b**) AFM image of Ag/AgClNPs–Liposomes–CTS (P6—Biohybrid III); magnification of the areas with “free” Ag/AgClNPs and Ag/AgClNPs bonded to liposomes are presented in the cropped orange and green fragments, respectively.

AFM images for biohybrid complexes II (P5) and III (P6) are demonstrated in Figure 6. In both cases, the presence of Ag/AgClNPs on the surface of the liposomes was detected (green crops from scan images). Obviously, the morphology of the liposomes is changed: they have an ellipsoidal shape with an apparent width (W, in its short axis) of ∼341 nm and length (L, in its long axis) of ∼516 nm for the P5 system, and W ≈ 480 nm and L ≈ 660 nm for the P6 system, while most liposomes in the AFM images of the P1 and P2 systems appear regularly spherical and not deformed (see Figure S5 in the Supplementary Materials).

The sizes of hybrid Ag/AgClNPs associated with liposomes (highlighted by circles in green crops) are 64–77 nm and 97–172 nm for biohybrids P5 and P6, respectively.

The average size of Biohybrid P5 obtained from the AFM image (Figure 6a) is 72.6 ± 18.2 nm, and this value is in good agreement with 76.3 nm obtained from SAXS (see Supplementary Materials).

A close examination of the area highlighted in orange in Figure 6b indicates the presence of “free” silver/silver chloride nanoparticles with an almost spherical shape and sizes between 42 and 78 nm, which is consistent with the values of 39.5 and 60.9 nm obtained from the SAXS method. The central NP has a dimension of 112 nm. Such larger nanoparticle sizes, from which SAXS scattering was not detected due to method limitations, are present over the entire studied area (Figure 6b, top image), and we assume they are nanoparticle aggregates.

#### 3.4.2. SEM Analysis of the Developed Bio-Based Materials

**Figure 7:**
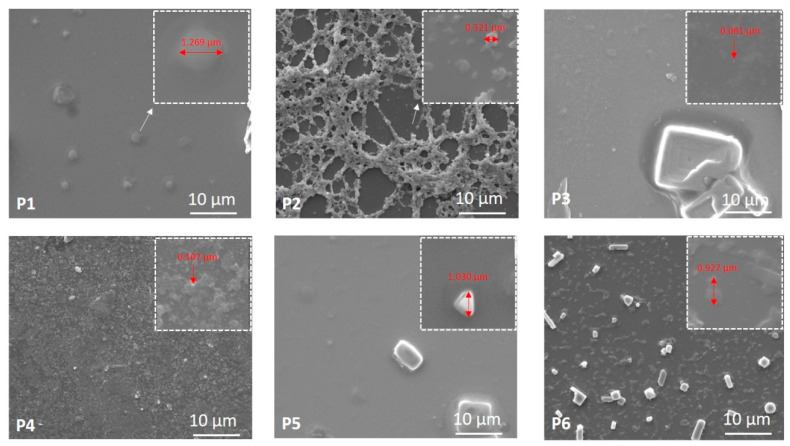
SEM images of the developed materials. Magnified SEM images of 3 × 3 µm^2^ are also presented, in which various particle sizes are measured.

Figure 7 presents the SEM images of Samples P1 up to P6 and a magnified area of 3 × 3 µm^2^. As one can observe, the samples are not identical in their morphologies. They present various structures with different shapes and sizes. For instance, Sample P1 shows individual spherical shape structures of lipid vesicles, whereas Sample P2 exhibits chain-like structures due to the presence of chitosan, including spherical structures in the mesh of the network. The silver nanoparticles without (Sample P3) and with (Sample P4) chitosan are spread all over the silicon plate surface. However, Sample P5 shows micrometric structures, and Sample P6 shows nano- and micrometric entities. This behavior can be explained by the diversity in the synthesis procedure of the samples, as explained in Table 1. As observed, the addition of chitosan resulted in increasing size of the samples, in good agreement with UV–vis absorption spectra, DLS results, SAXS data, and AFM analysis.

### 3.5. Mechanism of Biohybrid Formation

Summarizing our optical, structural, and morphological investigations, as well as the stability study of the biohybrid complexes and their components, a mechanism of Ag/AgClNP fabrication and biohybrid formation, can be discussed.

**Figure 8:**
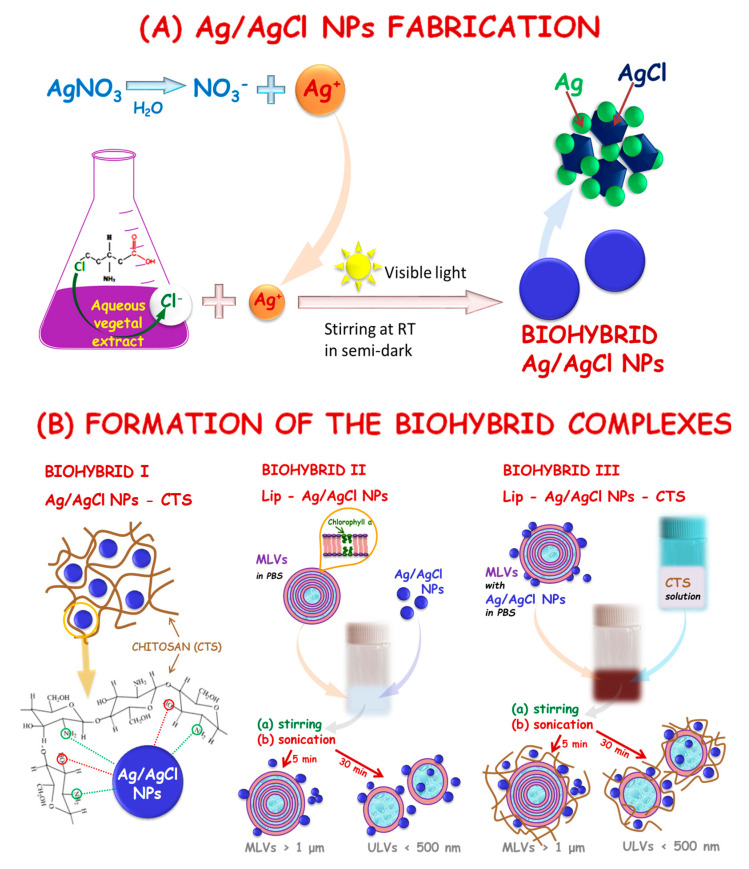
Mechanism of silver-based biohybrid formation.

Biohybrid Complex I (Ag/AgClNPs–CTS) with the proposed interaction model between the chitosan matrix and silver/silver chloride nanoparticles is shown schematically in Figure 8. The silver ions’ interaction takes place through the amino and hydroxyl groups of chitosan [43]. Moreover, it could be assumed that the formation of the chitosan-capped Ag/AgClNPs was confirmed by zeta potential measurements and by SAXS, SEM, and AFM studies.

Biohybrid II (Ag/AgClNPs–Lip) is a multilamellar vesicle (MLVs) with a surface coated with nanoparticles (Figure 8B). This output was obtained from a mixture of soybean lecithin liposomes and silver/silver chloride nanoparticles in PBS after sonication of the suspension for 5 min. Such a short time of treatment of the suspension with ultrasound does not lead to the formation of unilamellar vesicles (ULVs) and excludes with a high degree of probability the creation of other possible systems: (1) liposomes with bilayer-embedded nanoparticles, (2) liposomes with core-encapsulated nanoparticles, and (3) lipid-bilayer-coated nanoparticles. The obtained complex is a very stable system, as indicated by the zeta potential value of −33 mV. In addition, this value is very close to −31.7 mV for Ag/AgClNPs, and this result indicates the adhesion of nanoparticles on the liposome surface. At the same time, the interaction of Ag/AgClNPs with liposomes causes a change in liposome morphology. The ellipsoidal shape was observed by the AFM method, while the pure soybean lecithin liposomes were almost spherical, as clearly seen on the microscopic images (Figure S5a–c in the Supplementary Materials and Figure 6a). A similar effect was detected for Biohybrid III (Ag/AgClNPs–Lip–CTS): liposomes surrounded by chitosan have a spherical shape, and liposome–nanoparticles systems involved in the 3D chitosan network have an ellipsoidal shape (Figure S5d,e in the Supplementary Materials and Figure 6b). Even though this complex is less stable (ξ = +18.4 mV) than previously described, it has high antioxidant activity and antibacterial effectiveness, as will be further presented in Section 3.6.

### 3.6. Evaluation of Biological Activities of the Developed Bio-Based Materials

The in vitro bioactivities of materials phytogenerated from an aqueous extract of a mixture of nettle and grapes were tested by assessing: the antioxidant activity, the antibacterial properties against *S. aureus* and *E. coli*, the cytotoxicity, the antiproliferative activity, and the hemocompatibility. A therapeutic index was also calculated for each sample.

The biological performances of the developed materials were closely related to their zeta potential values, size, morphology, and structure.

**Figure 9:**
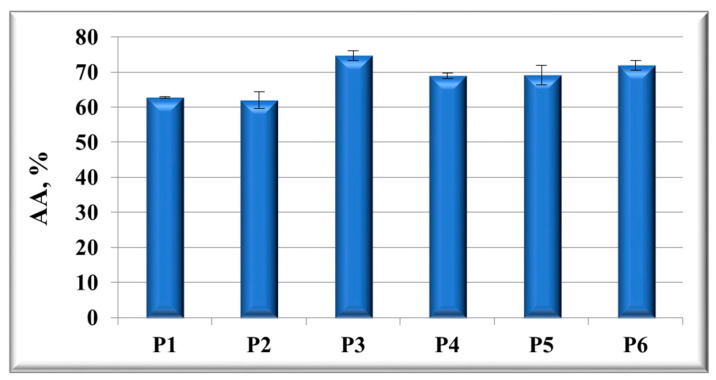
The in vitro antioxidant activity of the developed plasmonic composites and their “building blocks”, evaluated by the chemiluminescence method.

The samples showed good antioxidant activity, ranging from 62 to 75% (in vitro tested through the chemiluminescence method; see Figure 9).

The good effectiveness of Ag/AgClNPs-based materials in the scavenging activity of reactive oxygen species (ROS) is explained by: (i) their composition (especially the presence of “green” Ag/AgClNPs carrying antioxidant molecules such as polyphenols, chlorophyll, etc., arising from vegetal extracts) and (ii) their nanosized dimensions (which offer more reaction centers for ROS scavenging) [27].

**Figure 10:**
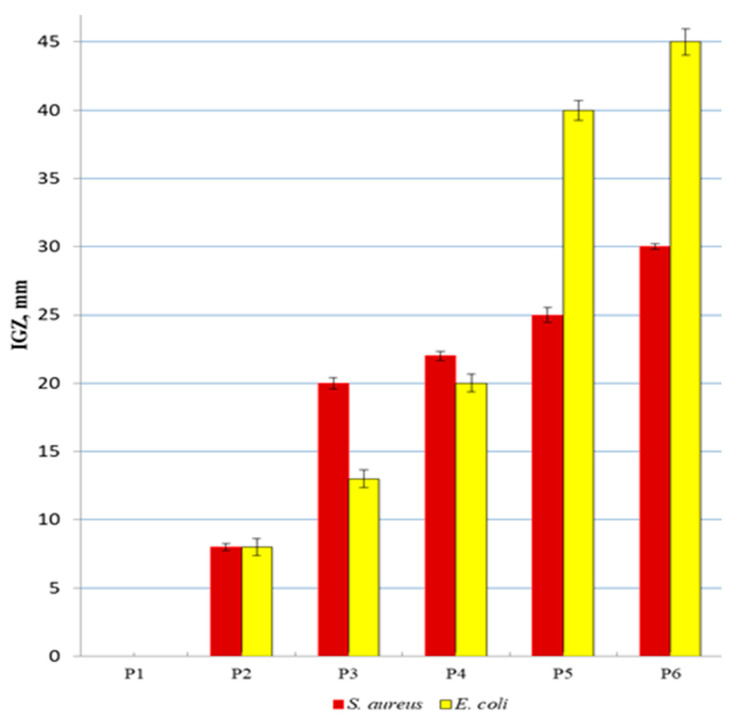
Antibacterial activity expressed as a diameter of the inhibition growth zone (IGZ) against *Staphylococcus aureus* ATTC 2592 and *Escherichia coli* ATCC 8738.

Antibacterial effectiveness of the developed biohybrids was tested against *Staphylococcus aureus* ATTC 2592 (as a representative Gram (+) bacteria) and *Escherichia coli* ATCC 8738 (as a representative Gram (-) bacteria) studied by the agar well diffusion method (see Figure 10).

The need for creating new antibacterial systems is an important research area in the medical field. *Staphylococcus aureus* and *Escherichia coli* are the most important human pathogens associated with nosocomial and community-acquired infections, causing serious health problems [44].

Our “green” obtained Ag/AgClNPs are more efficient against the two pathogens *S. aureus* and *E. coli* (IGZ *_S. aureus_* = 20 mm; IGZ *_E. coli_* = 13 mm) as compared to the AgClNPs prepared by Trinh et al. [45], who obtained IGZ values less than 11 mm for both strains. Kashyap et al. [20] prepared Ag/AgCl particles showing a zone of inhibition of 7.5 mm against *E. Coli*. Moreover, AgClNPs synthesized by Kota et al. [21] proved to be less potent against *S. aureus* (IGZ *_S. aureus_* = 18.5 mm) as compared to our silver nanoparticles.

The biohybrids containing “green” Ag/AgClNPs and Chla-Liposomes (Biohybrid II) exhibited high antibacterial properties against *Staphylococcus aureus* ATTC 2592 (IGZ = 25 mm) and *Escherichia coli* ATCC 8738 (IGZ = 40 mm) (see Sample P5 in Figure 10), as compared to Biohybrid I, which showed an IGZ value of 20 mm against *E. coli* and 22 mm against *S. aureus* (see Sample P4 in Figure 10).

Co-loading of phyto-derived Ag/AgClNPs, biomimetic membranes, and chitosan (Biohybrid III) resulted in impressive antibacterial effectiveness against *S. aureus* (IGZ = 30 mm) and *E. coli* (IGZ = 45 mm) (see Sample P6 in Figure 10), these remarkable properties being due to their composition.

These developed materials showed different behavior against two bacterial strains due to the differences in the bacterial cell wall structures. Gram (+) bacteria possess a thick peptidoglycan layer and no outer lipid membrane, while Gram (-) bacteria have an outer lipid membrane with pores and a unique periplasmic space with a thin peptidoglycan layer [46].

Direct contact of our developed materials with the bacterial cells resulting in perturbation/deterioration of cell walls and membranes leading to cell death is the most plausible antibacterial mechanism [47,48,49].

**Figure 11:**
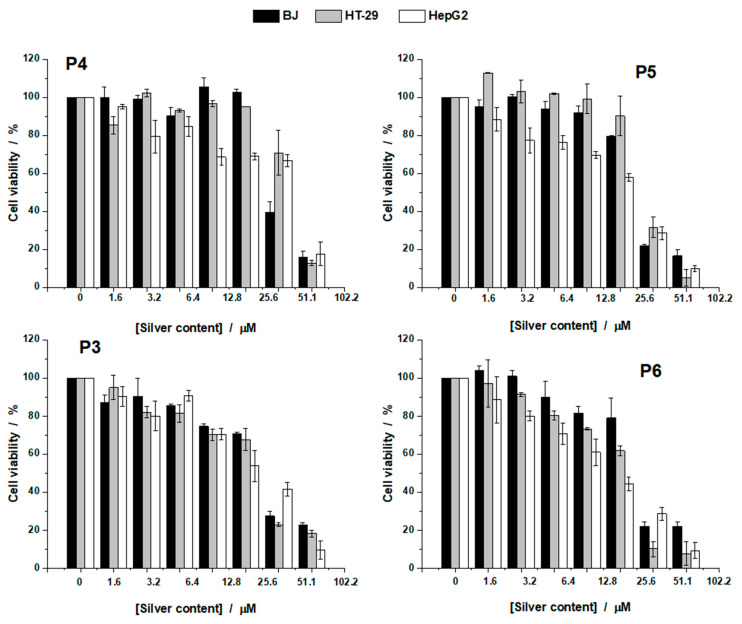
Cell viability of the silver-based materials (P3, P4, P5, and P6) against BJ, HT-29, and HepG2 cells after 24 h of treatment.

Cell viability results are reported in Figure 11 for all three cell lines investigated, highlighting the dependence of cell viability on the silver content and the type of cell line. Low doses of AgNPs had less cytotoxicity on the growth of normal cell lines (BJ cells), but enhanced cytotoxicity was observed with increasing doses. The results obtained are in correlation with those reported in the literature [50,51]. It was found that at concentrations less than 25.5 μM, the samples do not show a toxic effect, the cell viability being higher than 80% in the case of BJ cells. Chitosan addition to the AgNPs did not improve the efficiency of the NPs but only increased the overall concentration where the effect was observed. A better efficiency was observed for the samples prepared with the liposomes. Thus, the most efficient proved to be the NPs prepared with liposomes, P5 and P6, especially Sample P6, which, over the entire range, tested displayed less toxicity to normal cells and increased toxicity to cancer cells.

The values of IC_50_ and the therapeutic index (TI) are reported in Table 4. IC_50_ values were used further to calculate the TI, which is an indicator of the treatment efficiency against cancer cells. A value higher than one of TI indicates that the anticancer activity is higher compared with the cytotoxic activity of the samples investigated against the normal cells (BJ cells) [28].

From Table 4, one can see that Samples P3, P5, and P6 are more efficient against HepG2 cells, while P5 and P6 against HT-29 cells. The results indicated that Samples P3 and P4, which have in their composition only Ag and Ag and chitosan, respectively, are more toxic for normal cells as compared to cancerous cells. However, after the addition of lipids to the samples (P5 and P6), their efficacy increased, mainly by observing an increase in the toxicity against cancer cells.

**Figure 12:**
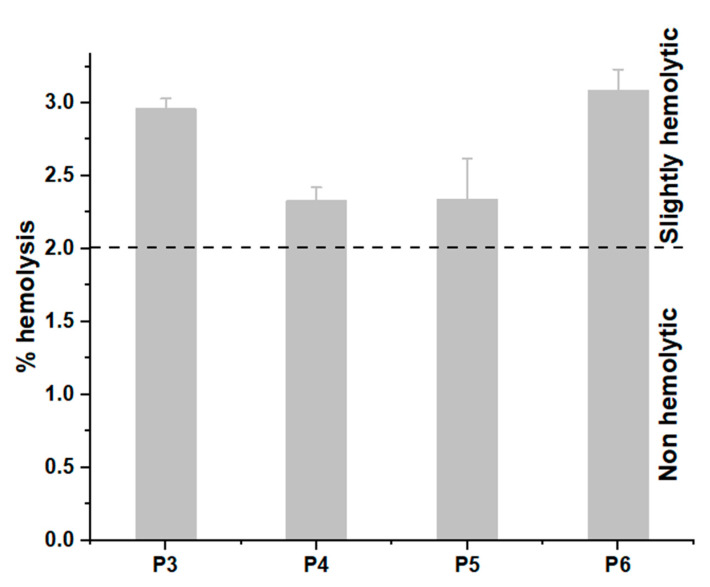
Hemolysis of the silver-based samples (P3, P4, P5, and P6) against hRBCs after 4 h of treatment at 102.2 µM.

Further, we investigated the effect induced by the highest concentration of the samples tested before against the red blood cells. At 102.2 µM, for each sample, the hemolysis percentage of new silver-based hybrids (Figure 12) is far below 5% [29], meaning that these samples are slightly hemolytic, but they will not be harmful when found in the bloodstream at smaller concentrations.

**Figure 13:**
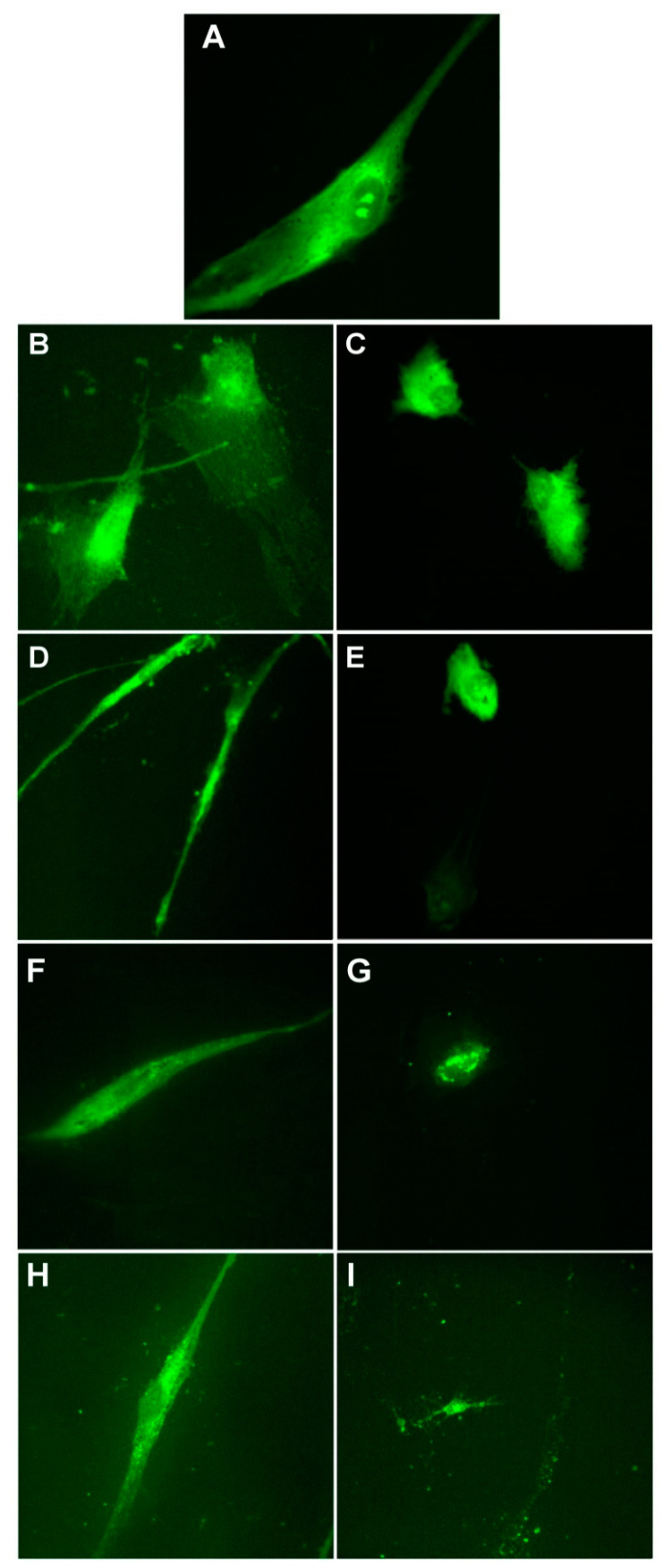
Morphology of control BJ cells (**A**) and cells after treatment with 6.4 µM and 51.1 µM of P3 (**B**,**C**), P4 (**D**,**E**), P5 (**F**,**G**), and P6 (**H**,**I**). Images are obtained with a 40× objective and using the epifluorescence mode.

**Figure 14:**
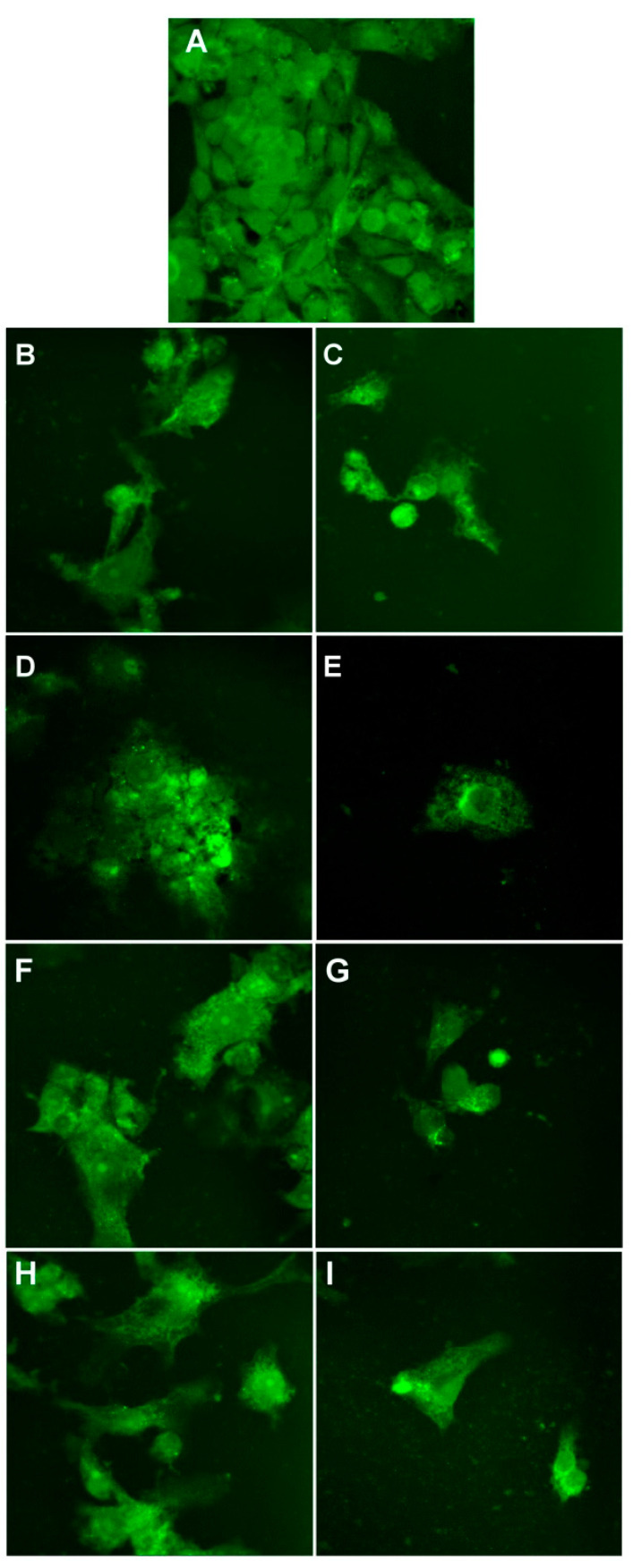
Morphology of control HT-29 cells (**A**) and cells after treatment with 6.4 µM and 51.1 µM of P3 (**B**,**C**), P4 (**D**,**E**), P5 (**F**,**G**), and P6 (**H**,**I**). Images are obtained with a 40× objective and in an epifluorescence mode.

**Figure 15:**
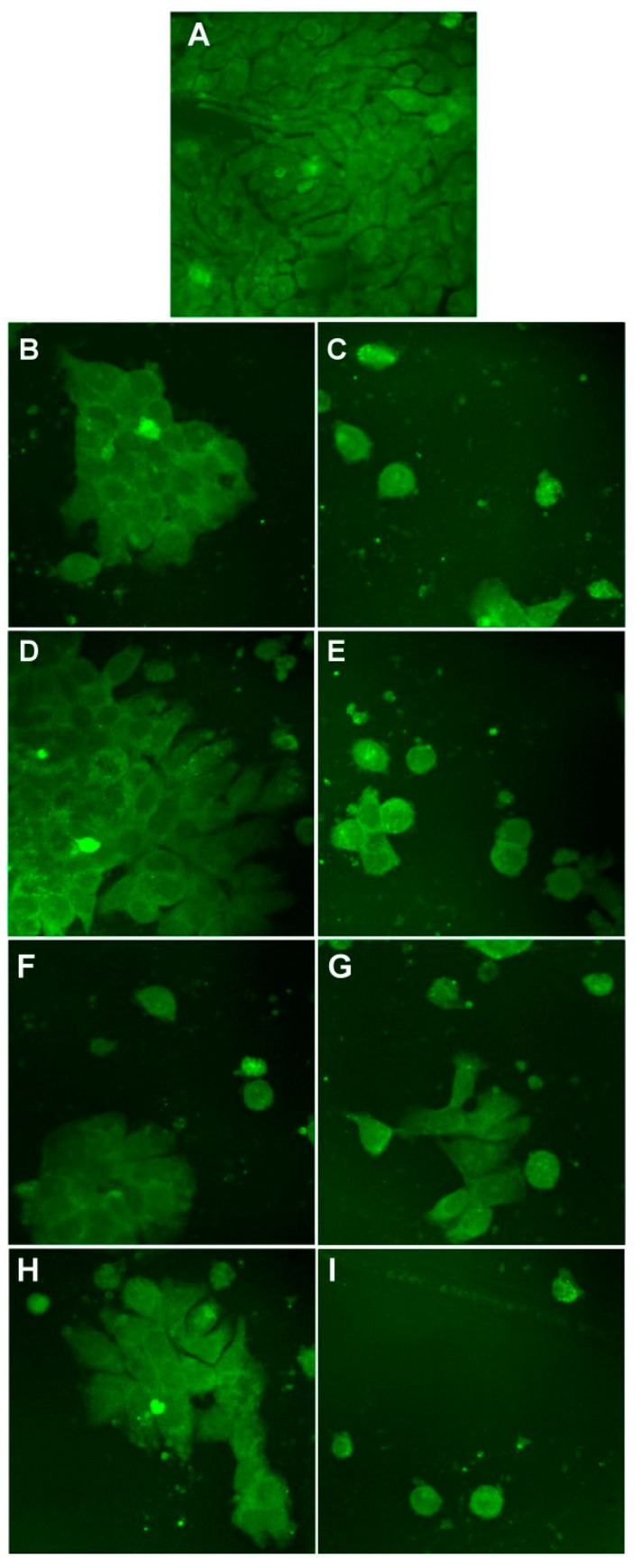
Morphology of control HepG2 cells (**A**) and cells after treatment with 6.4 µM and 51.1 µM of P3 (**B**,**C**), P4 (**D**,**E**), P5 (**F**,**G**), and P6 (**H**,**I**). Images are obtained with a 40× objective and in an epifluorescence mode.

Figure 13, Figure 14 and Figure 15 present the morphological changes induced by the treatment as compared to the control BJ cells. To determine how the presence of the NPs affects the morphology of the cells, we chose two different concentrations: a concentration not affecting the cells’ viability (6.4 µM) and a concentration above the IC_50_ values (51.1 µM). Similar concentrations were used for all the samples and all the cells that are further investigated.

The control BJ cells normally present an elongated shape and when treated with the smallest concentration of the sample, it could be observed that the morphology of these cells was not affected (Figure 13). However, at 51.1 µM silver content, the cells’ morphology was drastically affected: the cells’ branches were reduced, as well as the cell body. The results are in correlation with those reported for cell viability, where at 51.1 µM, the viability decreased by around 30–40% for all the samples investigated.

Figure 14 presents the morphological changes of HT-29 cells induced by different treatments. Control HT-29 cells grow in clusters as observed in Figure 14A. When treated with 6.4 µM, we can observe that the morphology of the cells was nonsignificantly affected. At 51.1 µM, the cells’ morphology is affected as well as the reduction of the number of cells.

In Figure 15 are presented the results for HepG2 cells. Control HepG2 cells grow in clusters, as observed in Figure 15A. When treated with 6.4 µM, we can observe that the morphology of the cells is not affected so much. However, at 51.1 µM, the number of cells is reduced. For both HT-29 and HepG2 cells, the results reported are in good concordance with the results reported for cell viability.

To summarize, the results obtained regarding the biological activity of the prepared materials are encouraging, taking into account that good bioperformances were obtained at a lower silver content compared to the data previously reported by our research group [10,27].

## 4. Conclusions

Vegetal wastes of grapes and nettle leaves were used to generate biohybrid entities by using the *Green Chemistry* principles.

The natural porphyrin chlorophyll *a*, inserted in biomimetic membranes, acted as a spectral sensor to monitor the generation of the biohybrids. The biointeraction between bionanometals and artificial cell membranes was detected by Chl*a* at the nano level, through changes in its spectral fingerprints.

The optical, structural, and morphological investigations correlated well, and the mechanism of Ag/AgClNP fabrication and biohybrid formation was discussed.

Microscopical investigations by AFM and SEM images gave information about the (quasi)spherical morphology and nanoscaled size of the samples.

XRD and SAXS investigations revealed the structural changes of the Ag/AgCl nanoparticles in the biohybrid complexes, highlighting the copresence of AgNPs and hybrid Ag/AgClNPs in the obtained silver-based systems. Particular attention was paid to the study of Biohybrid III (Ag/AgClNPs–Liposomes–CTS) because during the formation of biohybrid complexes, namely liposomes NPs, there is a high probability that small nanoparticles, such as AgNPs, may pass through the cell membranes, thereby affecting their functionality. However, in our case, it can be assumed that silver chloride nanoparticles (AgClNPs) or hybrid Ag/AgClNPs, being large in size, do not disturb the integrity of the membrane and accumulate on their outer leaflet, shown by the mechanism of biohybrid formation proposed by us.

Coincorporation of Chl*a*-labeled mimetic biomembranes, Ag/AgClNPs and chitosan, resulted in significant bioperformances. The obtained silver-based biohybrids showed good antioxidant properties by scavenging 71% of reactive oxygen species and also presented high antimicrobial activity against *Escherichia coli* (IGZ = 45 mm) and *Staphylococcus aureus* (IGZ = 30 mm). The silver/biomimetic membrane biohybrids with and without chitosan were hemocompatible and presented efficiency against HepG2 and HT-29 cancer cells. The therapeutic index of Ag/AgClNPs–Liposomes–CTS samples supports the antitumor effect; these biohybrids exhibited a TI value of 1.30 for HT-29 cancer cells and 1.77 for HepG2 cancer cells, highlighting their antitumor effect against two types of cell lines.

These results demonstrate that silver-containing hybrid entities could be used as building blocks to design new materials with many bioapplications, especially in the treatment of liver and colorectal cancer.

## Figures and Tables

**Table 1:** Codes and descriptions of the samples.

Sample Code	Description	C_Liposomes_ (mg/mL)	C_Ag_ (mM)	C_CTS_ (% *w*/*v*)
P1	Liposomes	0.34	-	-
P2	Liposomes—CTS	0.34	0	0.01
P3	Ag/AgClNPs	-	0.61	-
P4	Ag/AgClNPs—CTS (Biohybrid I)	-	0.61	0.01
P5	Ag/AgClNPs—Liposomes (Biohybrid II)	0.34	0.61	-
P6	Ag/AgClNPs–Liposomes–CTS (Biohybrid III)	0.34	0.61	0.01

**Table 2:** Mean sizes of crystallites (D_XRD_, nm) estimated from diffraction line broadening.

Silver-Based Samples’ Codes	Ag	AgCl	NaCl	SHPH
P3—Ag/AgClNPs	16	62	459	149
P4—Ag/AgClNPs–CTS	17	25	448	–
P5—Ag/AgClNPs–Lip	23	47	351	–
P6—Ag/AgClNPs–Lip–CTS	14	33	–	–

**Table 3:** Structural parameters derived from the analysis of SAXS data and the calculated diameter (D_SAXS_ = 2(5/3)^1/2^R_g_) of the Ag and Ag/AgCl nanoparticles for silver-based samples (P3–P6).

Silver-Based Samples’ Codes	R_g2_ (nm)	s_2_	m_2_	D_SAXS2_ (nm)	R_g1_ (nm)	s_1_	m_1_	D_SAXS1_ (nm)
P3—Ag/AgClNPs	33.7 ± 1.8	0.06 ± 001	2.2 ± 0.003	86.9 ± 1.8	10.1 ± 2.8	0.27 ± 0.08	4.2 ± 0.05	26.1 ± 2.8
P4—Ag/AgClNPs–CTS	35.3 ± 0.2	0.1± 0.03	2.9 ± 0.001	91.1 ± 0.2	12.4 ± 0.3	0.08 ± 0.02	4.1 ± 0.04	32.0 ± 0.3
P5—Ag/AgClNPs–Lip	43.4 ± 0.3	0.01 ± 0.005	2.5 ± 0.01	112.0 ± 0.3	15.7 ± 0.01	0.01 ± 0.03	4.1 ± 0.02	40.5 ± 0.01
P6—Ag/AgClNPs–Lip–CTS	23.6 ± 1.2	0.002 ± 0.0002	2.2 ± 0.02	60.9 ± 1.2	15.3 ± 0.7	0.01 ± 0.05	4.3 ± 0.05	39.5 ± 0.7

**Table 4:** IC_50_ and therapeutic index (TI) of the silver-based materials developed in our study.

	IC_50_			TI	
	BJ	HT-29	HepG2	HT-29	HepG2
P3	36.31	43.37	28.03	0.84	1.30
P4	49.31	56.26	50.04	0.88	0.99
P5	34.64	30.89	33.55	1.12	1.03
P6	35.7	27.52	20.15	1.30	1.77

## Data Availability

The data is included in the main text and/or the supplementary materials.

## Supplementary Materials

The following are available online at https://www.mdpi.com/article/10.3390/nano11071811/s1 at optical characterization (discussions; Figure S1. UV–vis absorption spectra of the developed materials; Figure S2. Fluorescence emission spectra of chlorophyll-labeled materials (λ_excitation_ = 430 nm); Figure S3. FTIR spectra; Particle size estimated by DLS measurements (Figure S4. Mean particle size (Zav, nm) and polydispersity index (PdI) of the samples, estimated by DLS measurements); SAXS comments—Guinier–Porod model; AFM discussions (Figures S5 and S6).
