# Supplementary material for: Biological Performances of Plasmonic Biohybrids Based on Phyto-Silver/Silver Chloride Nanoparticles
Source: Nanomaterials (Basel). 2021 Jul 12;11(7):1811. doi: 10.3390/nano11071811 (PMC8308221; doi:10.3390/nano11071811)
Supplement: Supplementary file 1 [file nanomaterials-11-01811-s001.zip › nanomaterials-1259149-supplementary.pdf]

nanomaterials

MDPI

Supplementary Materials

Biological Performances of Plasmonic Biohybrids Based on Phyto-Silver/Silver Chloride Nanoparticles

Yulia Gorshkova [1,2], Marcela-Elisabeta Barbinta-Patrascu [3,*], Gizo Bokuchava [1], Nicoleta Badea [4], Camelia Ungureanu [4], Andrada Lazea-Stoyanova [5], Mina Răileanu [3,6], Mihaela Bacalum [6], Vitaly Turchenko [1,7], Alexander Zhigunov [8] and Ewa Juszyńska-Gałązka [9]

[1] Joint Institute for Nuclear Research, Frank Laboratory of Neutron Physics, Joliot-Curie, 6, 141980 Dubna, Moscow Region, Russia; Yulia.Gorshkova@jinr.ru (Y.G.); gizo.bokuchava@jinr.ru (G.B.); turchenko@jinr.ru (V.T.)
[2] Institute of Physics, Kazan Federal University, 16a Kremlyovskaya Street, 420008 Kazan, Russia
[3] Department of Electricity, Solid-State Physics and Biophysics, Faculty of Physics, University of Bucharest, 405 Atomistilor Street, PO Box MG-11, 077125 Bucharest-Magurele, Romania; raileanumina@gmail.com
[4] General Chemistry Department, Faculty of Applied Chemistry and Materials Science, University "Politehnica" of Bucharest, 1-7, Polizu Street, 011061 Bucharest, Romania; nicoleta.badea@gmail.com (N.B.); ungureanucamelia@gmail.com (C.U.)
[5] National Institute for Lasers, Plasma and Radiation Physics, 409 Atomistilor Street, 077125 Magurele, Romania; andrada@infim.ro
[6] Department of Life and Environmental Physics, Institute for Physics and Nuclear Engineering, Horia Hulubei National, Reactorului, 30, 077125 Magurele, Romania; bmihaela@nipne.ro
[7] Department of Crystal Growth Laboratory, South Ural State University, 76, Lenin Aven., 454080 Chelyabinsk, Russia
[8] Institute of Macromolecular Chemistry AS CR, Heyrovskeho nam. 2, 162 06 Prague, Czech Republic; zhigunov@imc.cas.cz
[9] Institute of Nuclear Physics, Polish Academy of Sciences, ul. Radzikowskiego 152, 31-342 Krakow, Poland; ewa.juszynska-galazka@ifj.edu.pl
* Correspondence: marcela.barbinta@unibuc.ro

Citation: Gorshkova, Y.; Barbinta-Patrascu, M.-E.; Bokuchava, G.; Badea, N.; Ungureanu, C.; Lazea-Stoyanova, A.; Răileanu, M.; Bacalum, M.; Turchenko, V.; Zhigunov, A.; et al. Biological Performances of Plasmonic Biohybrids Based on Phyto-Silver/Silver Chloride Nanoparticles. *Nanomaterials* **2021**, *11*, 1811. https://doi.org/10.3390/nano11071811

Academic Editors: Henrich Frielinghaus and Lyudmila M. Bronstein

Received: 28 May 2021
Accepted: 8 July 2021
Published: 12 July 2021

Publisher's Note: MDPI stays neutral with regard to jurisdictional claims in published maps and institutional affiliations.

Copyright: © 2021 by the authors. Licensee MDPI, Basel, Switzerland. This article is an open access article distributed under the terms and conditions of the Creative Commons Attribution (CC BY) license (https://creativecommons.org/licenses/by/4.0/).

Optical Characterization

UV-Vis absorption spectra of developed materials (Figure S1) showed that the spectrum baseline position of liposomes, Ag/AgClNPs and Ag/AgClNPs/liposomes increased in height after addition of chitosan indicating an increase in size of these samples.

In the UV-Vis absorption spectrum of silver/silver chloride NPs (see sample P3), three main absorption peaks were identified: at 207 and 257 nm corresponding to the biomolecules (polyphenols, flavonoids, proteins, etc.) of the plant extract, and another one at 422 nm which is the SPR band characteristic for Ag/AgClNPs formation. After addition of chitosan to these nanoparticles, only two absorbances were observed: one at 250 nm (due to the presence of chitosan whose amino and hydroxyl groups interacted with the functional groups of biocompounds derived from vegetal matrix) and another one at 431 nm; this second peak is the SPR band which red shifted as a consequence of the interaction between Ag/AgClNPs and chitosan.

The spectral fingerprints of chlorophyll *a* and of silver nanoparticles were identified in the complex biohybrids P5 and P6. The height of the "main red" peak of Chla (at ~669 nm) decreased in the order: P1 > P2 > P5 > P6.

The fluorescence emission spectra of Chla-containing samples were investigated under 430 nm excitation wavelength. Figure S2 revealed the spectral signature of Chla on emission spectra of Chla-based samples: a strong and sharp emission peak located at 677 and 678 nm in Chla-labelled biomimetic membranes without chitosan and with chitosan, respectively. The fluorescence intensity dropped after chitosan addition. Fluores-

cence intensities decreased in the order: P1 > P2 > P5 > P6, this variation being in the same way as the height of the "main red" peak in UV-Vis absorption spectra of Chla incorporated into biomimetic membranes.

Fluorescently-labelled liposomes undergone considerably emission quenching after addition of phyto-Ag/AgClNPs ($\lambda_{excitation}$ = 430 nm), due to an energy-transfer or an electron-transfer process when the Chla porphyrinic ring located at the lipid bilayer/aqueous medium interface directly attach to the nanosilver surface [1]. Similar events were observed in our previous studies regarding biocomposites based on bio-AgNPs [2-4].

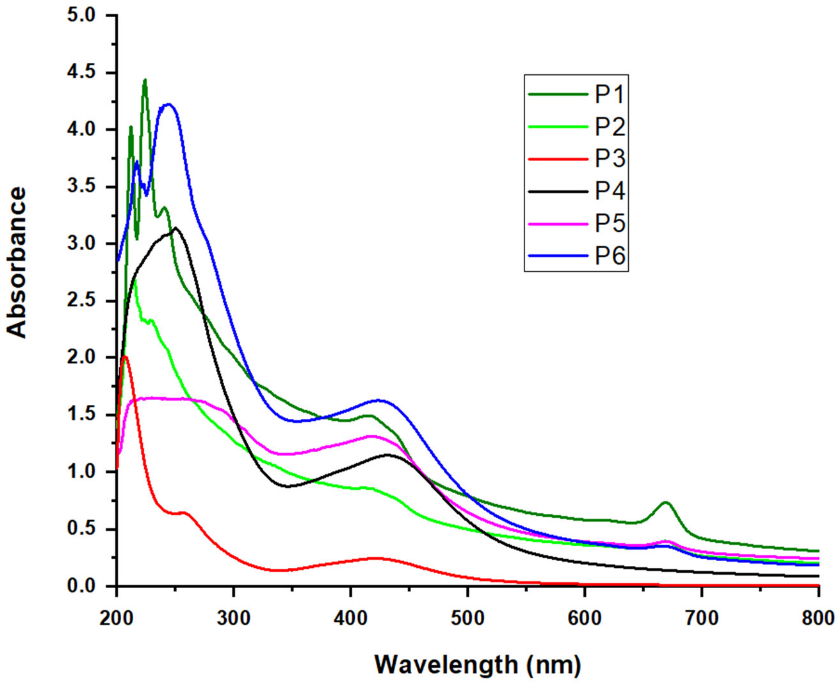

Figure S1. UV-Vis absorption spectra of developed materials.

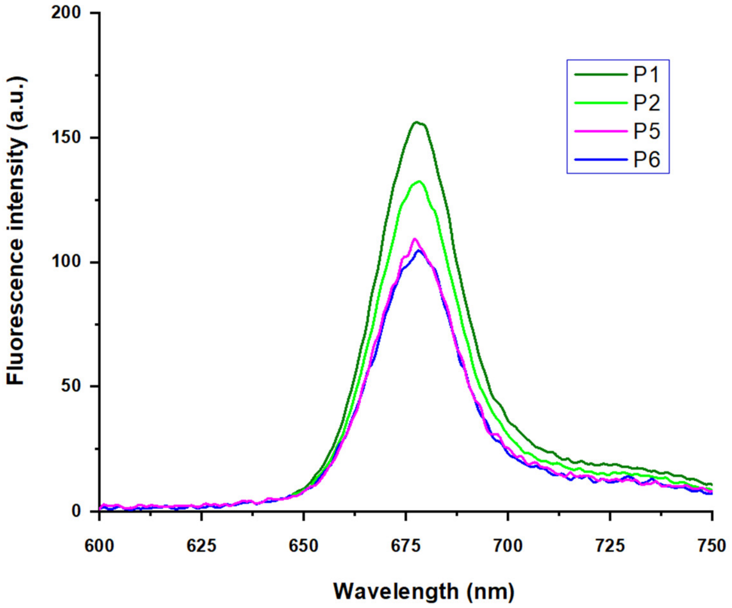

Figure S2. Fluorescence emission spectra of chlorophyll-labelled materials ($\lambda_{excitation}$ = 430 nm).

The formation of biohybrids were further confirmed by FTIR analysis (see Figure S3).

All tested mixtures contained phosphate buffer saline (PBS), which consists of Na_2HPO_4, KH_2PO_4, and NaCl. The vibration band for Na_2HPO_4 appears at ~1053 cm^{-1}, and conspicuous bands at 1240, 1160 and 1090 cm^{-1} [5], whereas for KH_2PO_4 we expect the vibration bands appear within the whole wavenumber region, i.e. from 700 to 1300 cm^{-1}, wherein the characteristic bands appear at 855 and 1074 cm^{-1}, and between 1275 and 1278 cm^{-1}.

FTIR spectrum of Chlorophyll *a* - labelled lecithin liposomes (sample P1, black line in Figure S3a, in the main text) demonstrates the contributions of all major lipid moieties, as expected for lipid molecular structure [4, 6]. The low wavenumber region of the spectrum (below 1800 cm^{-1}) is essentially related to the polar head groups of the lipids [6]. The peak from C=O corresponds to stretching bonds in ester groups at 1739 cm^{-1} [7, 8]. The vibration mode from C=C occurs at 1653 cm^{-1}. The hydrocarbon chains do contribute near 1464 cm^{-1} ($\delta(CH_2)$) and near 1375 cm^{-1} ($\delta(CH_3)$), while the phosphate contributions appear near 1240 cm^{-1} ($\nu_{as}(PO_2^-)$) and 1080 cm^{-1} ($\nu_s(PO_2^-)$). It could be also observed the peaks at 1170 cm^{-1} and 976 cm^{-1}, which correspond to vibration modes of ν_{as}(C-O) and ($\nu_{as}(N^+(CH_3)_3)$), respectively [4]. The peak at 860 cm^{-1} is associated with distortive vibrations of the NH_2 group of primary and secondary amines. Broad bands occurring in the frequency range of 660 – 910 cm^{-1}, were observed for all substances under study. The most prominent contribution of the lipid hydrocarbon chains appear in the second spectral region between 3050 and 2800 cm^{-1}. The symmetrical and asymmetrical CH stretching band in the CH_2 groups of the alkyl chains of the lipids were observed around 2855 and 2922 cm^{-1}, respectively [6, 9]. The broad band at 3300 cm^{-1} corresponds to the stretching vibrations of N-H and O-H groups and intermolecular hydrogen bonds.

FTIR spectra of Ag/AgClNPs (P3, blue line in Figure S3a, in the main text) exhibited prominent peaks of organic molecules. The band at 1636 cm^{-1} denotes the presence of the alkene C=C stretching vibrations in the plant extract [10, 11]. The peak at 1360 cm^{-1} representing the C–O stretching vibration in flavonoids [12]. The broad band around 3295 cm^{-1} is responsible for the O-H stretching of alcohol group present in plant extract [13].

After addition of chitosan, the medium bands attributed to antisymmetric stretching of C–O group of polysaccharides and/or chlorophyll [14] shifted from 1070 cm^{-1} (in sample P3) to 1076 cm^{-1} (in sample P4).

The complex system of Ag/AgClNPs–Liposomes (P5, Figure S3a-b) collected all the bands that we observed only for liposomes, or only for silver nanoparticles.

The broad bands at 3295 cm^{-1} in all the samples indicate the presence of hydroxy group, H-bonded –OH stretch [8].

As observed, after addition of chitosan to samples P1, P3 and P5, an increase in intensity of IR bands should be noted in all cases.

In Figure S3b, some events occurred after CTS addition to the sample P5, as follows:

- The band corresponding to the vibrations of –OH, –CH in the ring [8] located at 1357 cm^{-1} (sharp band with a shoulder at 1374 cm^{-1}) in P5 shifted to 1370 cm^{-1} (with a shoulder at 1394 cm^{-1}) when chitosan was added. The –OH groups arise from phenols of plant extracts.
- Other bands (1610–1550 & 1425–1300 cm^{-1}) associated with the carboxylate and hydroxyl compounds (phenols or alcohols) [8] overlapped and tend to be less pronounced, and slightly shifted in P6.
- Some bands attributed to carbonyl compound group frequencies at ~1770-1790 cm^{-1} [8] overlapped in P6.

These findings indicate the involvement of hydroxyl and carbonyl groups (belonging to polyphenols and proteins, respectively) in generation of biohybrids P5 and

P6.

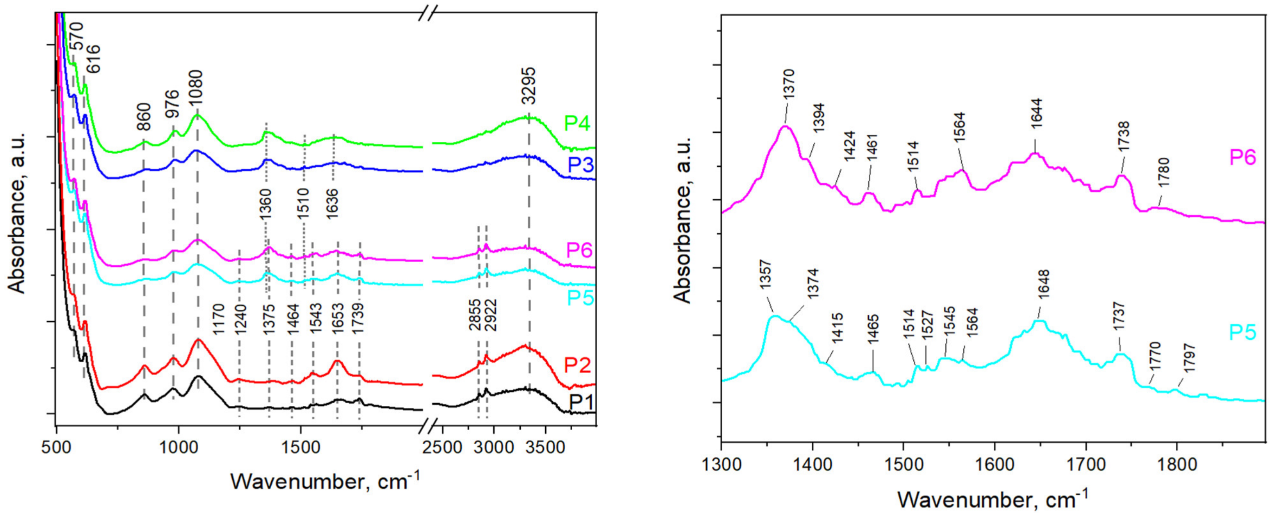

(**a**) (**b**)

Figure S3. (**a**) FTIR spectra of initial components and its biohybrid nanocomplexes in PBS buffer at temperature of 40°C: P1 – Liposomes, P2 – Liposomes–CTS, P3 – Ag/AgClNPs, P4 – Ag/AgClNPs–CTS, P5 – Ag/AgClNPs–CTS–Liposomes, P6 – Ag/AgClNPs–Liposomes–CTS. (**b**) Comparative presentation of FTIR spectra of P5 and P6, in the wavenumber region of 1300-1800 cm^{-1}.

Particle size estimated by DLS Measurements

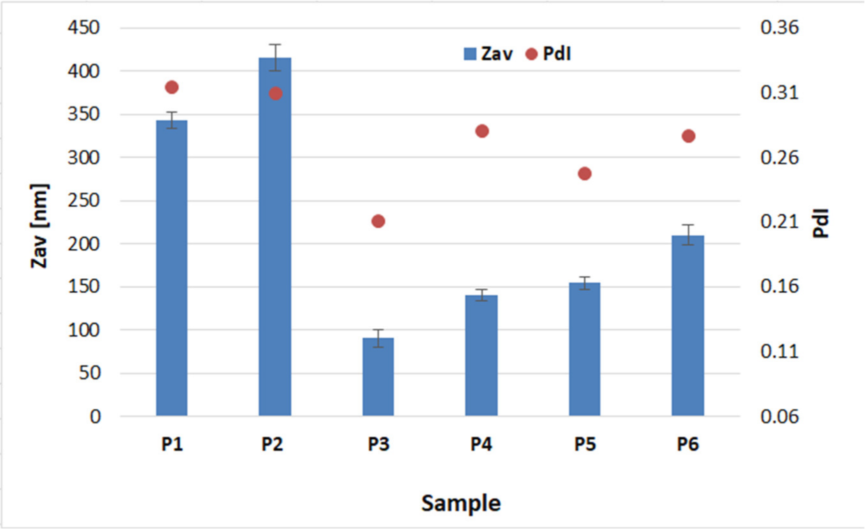

Figure S4. Mean particle size (Zav, nm) and polydispersity index (PdI) of the samples, estimated by DLS measurements.

SAXS Comments- Guinier-Porod Model

Despite the low concentration used, experiments became successful due to high contrast between silver/ silver chloride and solvent, as intensity $I(Q) \sim (\bar{\rho}_e - \rho_{s,e})$. The estimated values of scattering length density (SLD) for given SAXS setup are as follows: $\varrho_{Ag,e} = 77.9 \cdot 10^{-6}$Å^{-2}, $\varrho_{AgCl,e} = 42.5 \cdot 10^{-6}$Å^{-2}, $\varrho_{s,e} = 9.47 \cdot 10^{-6}$Å^{-2}. In addition, the SAXS results are less sensitive to the other components of the investigated systems due to the much lower contrast. The estimated SLD values for chitosan ($\varrho_{CTS,e}$) can be taken in range $(1.36 \div 2.73) \cdot 10^{-6}$Å^{-2} for density of $((C_6H_{11}NO_4)_n)$ in range of 0.15–0.3 g/cm^3. Also, we can neglect the contribution of the scattering on the liposomes, as it is quite poor in our systems due to extremely low concentration (C_{lip} = 0.33 mg/mL).

As scattering curves are mostly featureless, a generalized Guinier-Porod model was used to extract the information about the shape and size of the Ag/AgCl NPs. For i-*th* level, this empirical model has the form [15]:

$$I(Q) = \begin{cases} G_i\, Q^{-s_i} \exp\left(-\frac{R_{g_i}^2 Q^2}{3-s_i}\right) & Q \leq Q_i^* \\ D_i Q^{-m_i} & Q \geq Q_i^* \end{cases}, \quad (1)$$

where G_i is the exponential (Guinier) prefactor, s_i is a dimension variable, R_{g_i} is a Radius of gyration, m_i is a Porod exponent.

The prefactor D_i and the quantity Q_i^* are obtained from the continuity conditions of the Guinier and Porod terms as well as of their derivatives. They can be written explicitly as:

$$Q_i^* = \frac{1}{R_{g_i}} \sqrt{(m_i - s_i)(3 - s_i)/2}, \quad (2)$$

and, respectively:

$$D_i = G_i\, exp\left(-\frac{R_{g_i}^2 Q^2}{3-s_i}\right) (Q_i^*)^{m_i - s_i}, \quad (3)$$

The first term in Eq. 1 is the generalized Guinier law, which is determining method for calculating the sizes of scattering objects of any shape in the Q-region satisfying the condition: ($QR_g < 1$). For 3D globular objects (such as spheres), s = 0 and one recovers the standard Guinier formula. For 2D symmetry (such as for rods) s = 1, and for 1D symmetry (such as for lamellae or platelets) s = 2 [16, 17]. The fractal dimension, D^m, and equilibrium structure qualities were evaluated from a relatively larger Q region using the Porod low – the second term in Eq. 1.

AFM Discussions

The sonication time effect on liposome formation was observed. It is well known that sonication is an effective method for producing single-layer liposomes, called unilamellar vesicles (ULVs). Thus, very small ULVs with almost spherical shape and an average size of 43.8±6.3 nm, as well as ULVs in the range between 65 and 150 nm (Figure S5.a), are formed by ultrasonic treatment of the initial solution (P1 system – multilamellar vesicles (MLVs) in PBS) about 30 minutes or more. At the same time, liposomes surrounded by chitosan have sizes ranging from 50 to 300 nm (Figure S5.d). Increasing the size of observed objects is caused by successful surface chitosan modification of the pure liposomes [18-20].

Biohybrids II and III are a combination of the two or three components mentioned above. For clear microscopic visualization of all entities, the liposomes and Ag/AgClNPs should have dimensions that differ by an order. For this purpose, the sonication time was reduced to 20 min. In this case, small ULVs are not observed, and soybean lecithin liposomes have a size of at least 100 nm (Figure S5.b), while the sizes of hybrid silver/silver chloride nanoparticles remain the same.

Our results for pure liposomes and liposomes with chitosan shell showed spherical-like particles with a smooth surface in case of the P1 system (Figure S5.c) and with a rough surface in case of the P2 system (Figure S5.e), suggesting that sample preparation and AFM scanning did not disrupt the observed objects.

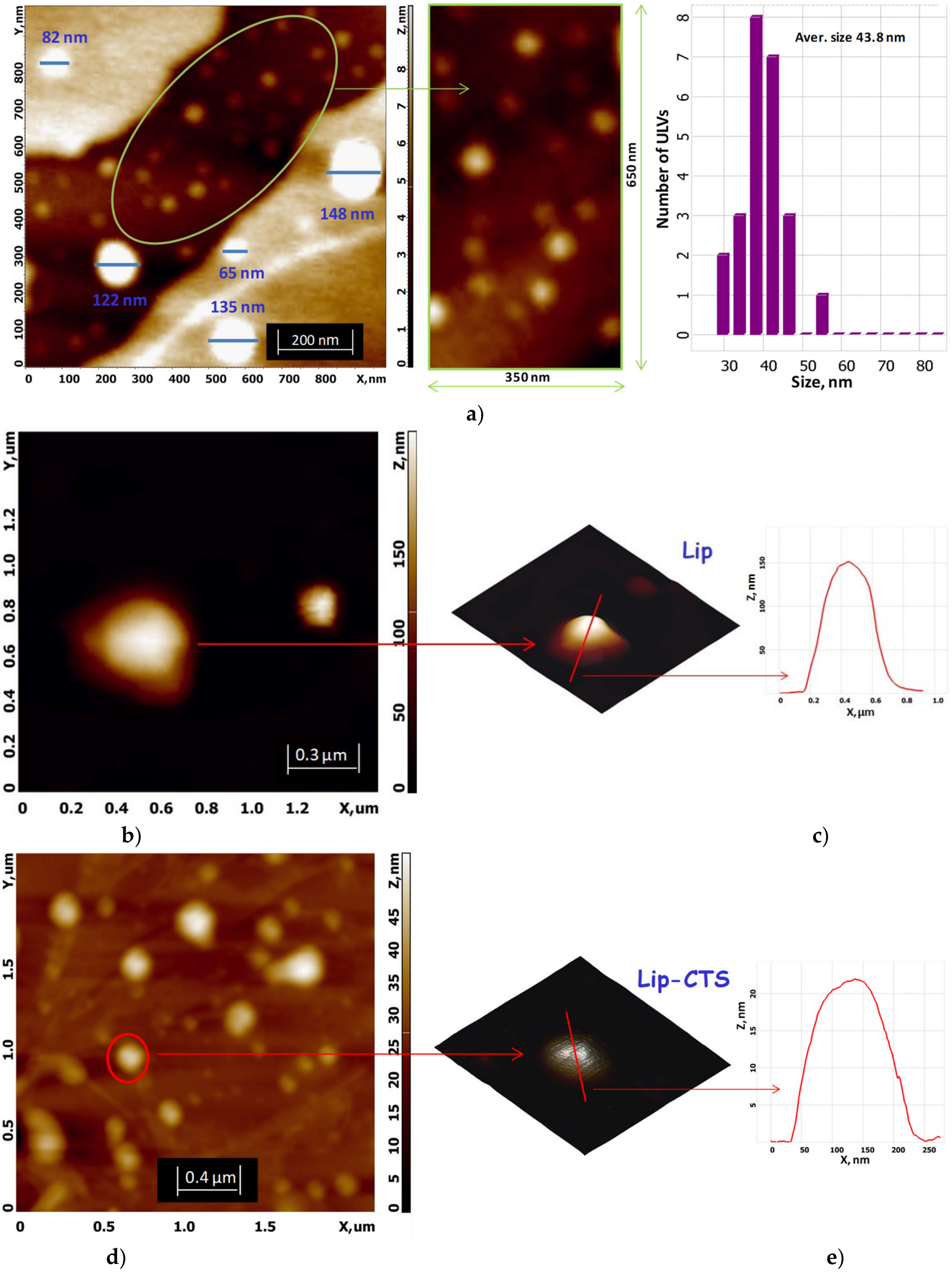

Figure S5. AFM images for the P1 system (chlorophyll a–labelled soybean lecithin liposomes): **a**) for liposomes sonicated for 30 minutes (from left to right: 1 µm x 1 µm scanned image, cropped image of the selected area and ULVs size distribution for this area) and **b**) for liposomes sonicated for 20 minutes, **c**) 3D image and height profile of single liposome. **d**) – AFM image for the P2 system (chlorophyll a–labelled soybean lecithin liposomes with chitosan) treated with ultrasound for 30 minutes; **e**) 3D zoomed detail (460 nm x 460 nm and z axis from 0 to 24.6 nm scales) and height profile of only one liposome.

Another important question on the formation of biohybrids II and III, namely, whether Ag/AgCl nanoparticles can penetrate inside liposomes under the action of ultrasound, has not yet been discussed. Attempt to resolve this issue was done for Ag/AgClNPs–Lip–CTS biohybrid complex. It is well known that long scanning times when performing AFM analysis causes liposome collapse and the formation of a concave structure with a compressed central part. For example, Egg PC liposomes with "concave shape" characterized by a depressed central part and a higher outline were observed by AFM after 2 h of scanning [21]. The oblate liposomes in our study were stable for a long time and only 2 days after their adsorption to mica substrate, drying and keeping at room temperature, we observed the spontaneous liposome collapse and formation of the concave structure as shown for biohybrid III (Figure S6). Hybrid Ag/AgClNPs in this case, on the contrary, remain stable with non-deformed spherical shape and have a lower tendency to aggregation, as was previously found for freshly prepared biohybrid III (see Figure 6b, in the main text).

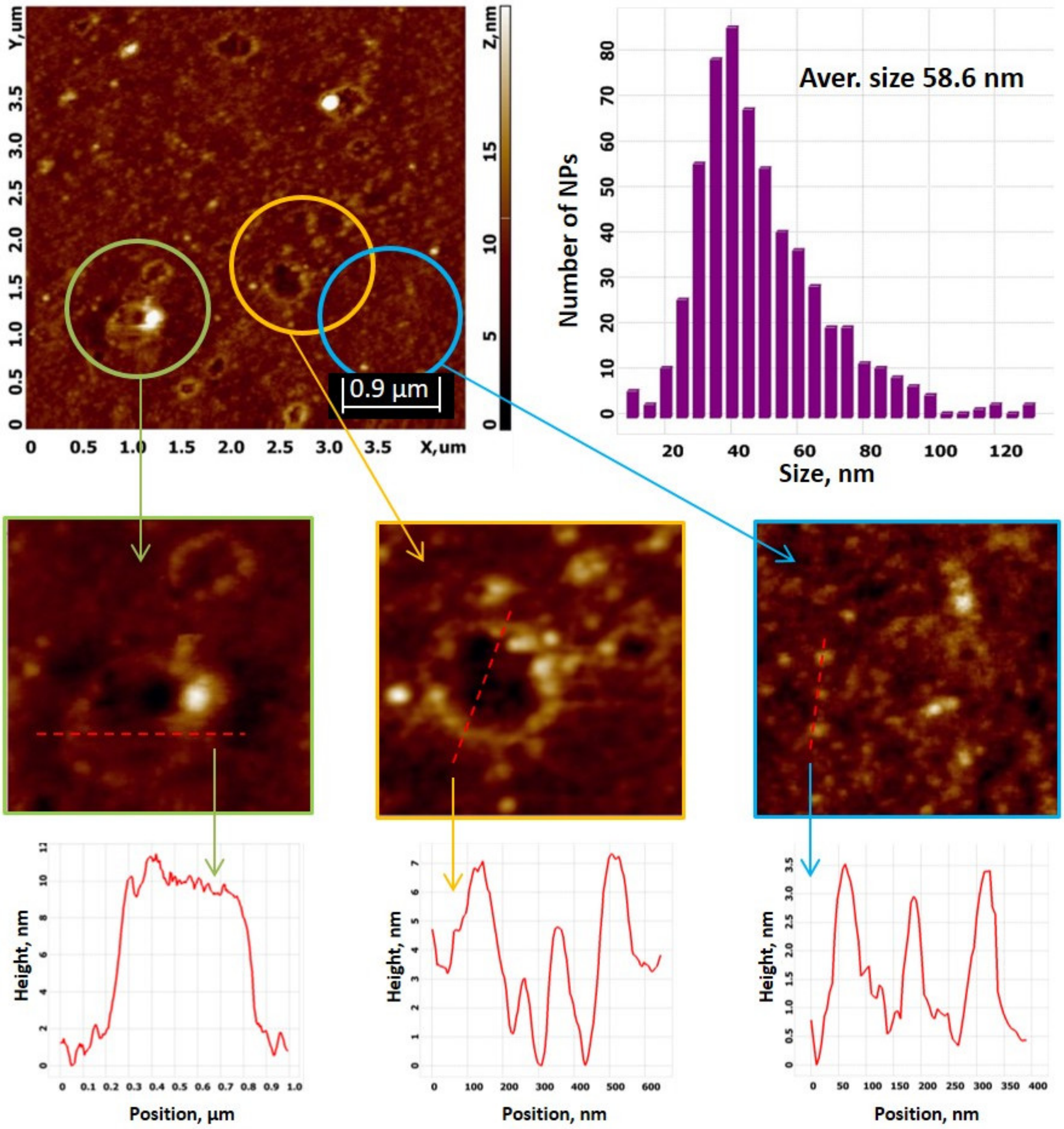

Figure S6. AFM topology of Ag/AgClNPs–Lip–CTS biohybrid scanned 2 days after their preparation with detailed information: size distribution of the Ag/AgClNPs; magnified images and height profiles for partially and fully collapsed liposomes, and for Ag/AgClNPs.

The average size of Ag/AgCl nanoparticles coated with chitosan, determined from the particle size distribution over the entire scanning area (Figure S6), excluding the contribution from liposomes, is 58.6±22.5 nm. This value is very close to 56.9 nm for Ag/AgClNPs coated with chitosan (P4 – biohybrid I, see Figure 5, right, in the main text). Additionally, the height of hybrid nanoparticles is almost constant value, as follows from the height profile for a random direction in the region where only Ag/AgClNPs are observed (Figure S6), image with blue frame) and the previous height analysis for the P3 and P4 systems. Two magnified images with green and orange frames (Figure S6) show partially and completely collapsed liposomes, respectively. The two middle peaks in the height profile for liposome with concave structure can be attributed to Ag/AgClNPs, and these particles could be located both inside and on the surface of fresh liposomes. However, the number of these particles is very limited (2 – 5 per liposome) and we concluded that hybrid Ag/AgClNPs are predominantly localized at the surface of liposomes in biohybrid III.

References

1. Falco, W.F.; Queiroz, A.M.; Fernandes, J.; Botero, E.R.; Falcao, E.A.; Guimarães, F.E.G.; M'Peko, J.-C.; Oliveira, S.L.; Colbeck, I.; Caires, A.R.L. Interaction between chlorophyll and silver nanoparticles: a close analysis of chlorophyll fluorescence quenching. *J. Photochem. Photobiol. A* **2015**, 299, 203–209. https://doi.org/10.1016/j.jphotochem.2014.12.001
2. Barbinta-Patrascu, M.E.; Badea, N.; Pirvu, C.; Bacalum, M.; Ungureanu, C.; Nadejde, P.L.; Ion, C.; Rau, I. Multifunctional soft hybrid bio-platforms based on nano-silver and natural compounds. *Mat. Sci. Eng. C* **2016**, 69, 922-932. doi:10.1016/j.msec.2016.07.077
3. Barbinta-Patrascu, M. E.; Badea, N.; Bacalum, M.; Antohe, S. Novel bio-friendly nanomaterials based on artificial cell membranes, chitosan and silver nanoparticles phytogenerated from *Eugenia caryophyllata* buds: eco-synthesis, characterization and evaluation of bioactivities. *Rom.Rep.Phys*. **2020**, 72(1), 601.
4. Barbinta-Patrascu, M.E.; Badea, N.; Bacalum, M.; Ungureanu, C.; Suica-Bunghez, I.R.; Iordache, S.M.; Pirvu, C.; Zgura, I.; Maraloiu, V.A. 3D hybrid structures based on biomimetic membranes and *Caryophyllus aromaticus* - "green" synthesized nano-silver with improved bioperformances, *Mat. Sci. Eng. C* **2019,** 101, 120-137**.** DOI: 10.1016/j.msec.2019.03.069 ; https://doi.org/10.1016/j.msec.2019.03.069
5. Mello, M.L.S.; Vidal, B.C. Changes in the Infrared Microspectroscopic Characteristics of DNA Caused by Cationic Elements, Different Base Richness and Single-Stranded Form. *PLoS ONE* **2012**, 7(8), e43169. doi:10.1371/journal.pone.0043169
6. Derenne, A.; Claessens, T.; Conus, C.; Goormaghtigh, E. Infrared spectroscopy of membrane lipids. In: *Encyclopedia of Biophysics*; Roberts, G.C.K., Ed.; Springer-Verlag: Berlin/Heidelberg, Germany, 2013; pp. 1074–1081.
7. Trinh, N.D.; Nguyen, T.T.B.; Nguyen, T.H. Preparation and characterization of silver chloride nanoparticles as an antibacterial agent. *Adv. Nat. Sci: Nanosci. Nanotechnol.* **2015**, 6(4), 045011. doi:10.1088/2043-6262/6/4/045011
8. Coates, J. Interpretation of Infrared Spectra, a Practical Approach. In *Encyclopedia of Analytical Chemistry*; Meyers, R.A., Ed.; JohnWiley & Sons Ltd.: Chichester, UK, 2000.
9. Mehrotra, R. Infrared Spectroscopy, Gas Chromatography/Infrared in Food Analysis. In *Encyclopedia of Analytical Chemistry*, Meyers, R.A., Ed.; John Wiley & Sons, Ltd., USA, 2006; doi:10.1002/9780470027318.a1013
10. Khattat, M.; Mohammad, S.; Mehrnaz, K.; Amir, R. A comparative study of stability, antioxidant, DNA cleavage and antibacterial activities of green and chemically synthesized silver nanoparticles. *Artif. Cells Nanomed. Biotechnol.* **2018**, 46, 1-10. Doi:10.1080/21691401.2018.1527346
11. Gannimani, R.; Perumal, A.; Krishna, S.B.; Muthusamy, S.K.; Mishra, A.; Govender, P. Synthesis and antibacterial activity of silver and gold nanoparticles produced using aqueous seed extract of *Protorhus longifolia* as a reducing agent. Dig. J. Nanomater. Biostructures **2014**, 9(4), 1669-1679.
12. Pawlikowska-Pawlęga, B.; Misiak, L.E.; Zarzyka, B.; Paduch, R.; Gawron, A.; Gruszecki, W.I. Localization and interaction of genistein with model membranes formed with dipalmitoylphosphatidylcholine (DPPC). *Biochim. Biophys. Acta* **2012**, 1818, 1785–1793. https://doi.org/10.1016/j.bbamem.2012.03.020
13. Kasturi, J.; Veerapandian, S.; Rajendiran, N. Biological synthesis of silver and gold nanoparticles using apiin as a reducing agent. *Colloids Surf. B Biointerfaces* **2009**, 68(1), 55-60. doi:10.1016/j.colsurfb.2008.09.021
14. Balachandran, Y.L.; Girija, S.; Selvakumar, R.; Tongpim, S.; Gutleb, A.C.; Suriyanarayanan, S. Differently environment stable bio-silver nanoparticles: Study on their optical enhancing and antibacterial properties. *PLoS ONE* **2013**, 8(10), e77043. http://dx.doi.org/10.1371/journal.pone.0077043
15. Hammouda, B. A new Guinier-Porod model. *J. Appl. Cryst*. **2010**, 43, 716-719. https://doi.org/10.1107/S0021889810015773
16. Hjelm Jnr, R.P.; Thiyagarajan, P.; Sivia, D.S.; Lindner, P.; Alkan, H.; Schwahn, D. Small-angle neutron scattering from aqueous mixed colloids of lecithin and bile salt. *Prog. Colloid Polym. Sci.* **1990**, 81, 225–231. DOI: 10.1007/bfb0115557

17. Hjelm Jnr, R.P., Thiyagarajan, P.; Alkan, H. A small-angle neutron scattering study of the effects of dilution on particle morphology in mixtures of glycocholate and lecithin. *J. Appl. Cryst*. **1981**, 21, 858–863. DOI: 10.1107/S002188988800531X
18. Pu, C.; Tang, W. A chitosan-coated liposome encapsulating antibacterial peptide, Apep10: characterisation, triggered-release effects and antilisterial activity in thaw water of frozen chicken. *Food & Function* **2016**, 7(10), 4310–4322. doi:10.1039/c6fo00858e
19. Shin, G.H.; Chung, S.K.; Kim, J.T.; Joung, H.J.; Park, H.J. Preparation of Chitosan-Coated Nanoliposomes for Improving the Mucoadhesive Property of Curcumin Using the Ethanol Injection Method. *J. Agr. Food Chem*. **2013**, 61, 11119-11126. doi:10.1021/jf4035404
20. Phetdee, M.; Polnok, A.; Viyoch, J. Development of chitosan-coated liposomes for sustained delivery of tamarind fruit pulps extract to the skin. *Int. J. Cosmetic Sci*. **2008**, 30(4), 285-295. doi:10.1111/j.1468-2494.2008.00447.x
21. Ruozi, B.; Tosi, G.; Leo, E.; Vandelli, M.A. Application of atomic force microscopy to characterize liposomes as drug and gene carriers, *Talanta* **2007**, 73(1), 12-22. https://doi.org/10.1016/j.talanta.2007.03.031
